# Silencing PinX1 enhances radiosensitivity and antitumor-immunity of radiotherapy in non-small cell lung cancer

**DOI:** 10.1186/s12967-024-05023-y

**Published:** 2024-03-02

**Authors:** Jieping Qiu, Ying Xia, Yawei Bao, Jingjing Cheng, Lei Liu, Dong Qian

**Affiliations:** 1 Present Address: Division of Life Sciences and Medicine, Department of Radiation Oncology, The First Affiliated Hospital of USTC, University of Science and Technology of China, Hefei, 230001 China; 2 Core Facility Center for Medical Sciences, The First Affiliated Hospital of USTC (Anhui Provincial Hospital), Hefei, 230001 Anhui China

**Keywords:** Non-small cell lung cancer, Radiosensitivity, Radiotherapy, Immunotherapy, Telomere dysfunction

## Abstract

**Background:**

We aimed to investigate the effects of PinX1 on non-small cell lung cancer(NSCLC) radiosensitivity and radiotherapy-associated tumor immune microenvironment and its mechanisms.

**Methods:**

The effect of PinX1 silencing on radiosensitivity in NSCLC was assessed by colony formation and CCK8 assay, immunofluorescence detection of γ- H2AX and micronucleus assay. Western blot was used to assess the effect of PinX1 silencing on DNA damage repair pathway and cGAS-STING pathway. The nude mouse and Lewis lung cancer mouse model were used to assess the combined efficacy of PinX1 silencing and radiotherapy in vivo. Changes in the tumor immune microenvironment were assessed by flow cytometry for different treatment modalities in the Lewis luuse model. The interaction protein RBM10 was screened by immunoprecipitation-mass spectrometry.

**Results:**

Silencing PinX1 enhanced radiosensitivity and activation of the cGAS-STING pathway while attenuating the DNA damage repair pathway. Silencing PinX1 further increases radiotherapy-stimulated CD8^+^ T cell infiltration and activation, enhances tumor control and improves survival in vivo; Moreover, PinX1 downregulation improves the anti-tumor efficacy of radioimmunotherapy, increases radioimmune-stimulated CD8^+^ T cell infiltration, and reprograms M2-type macrophages into M1-type macrophages in tumor tissues. The interaction of PinX1 and RBM10 may promote telomere maintenance by assisting telomerase localization to telomeres, thereby inhibiting the immunostimulatory effects of IR.

**Conclusions:**

In NSCLC, silencing PinX1 significantly contributed to the radiosensitivity and promoted the efficacy of radioimmunotherapy. Mechanistically, PinX1 may regulate the transport of telomerase to telomeres through interacting with RBM10, which promotes telomere maintenance and DNA stabilization. Our findings reveal that PinX1 is a potential target to enhance the efficacy of radioimmunotherapy in NSCLC patients.

**Supplementary Information:**

The online version contains supplementary material available at 10.1186/s12967-024-05023-y.

## Background

Non-small cell lung cancer (NSCLC) accounts for roughly 85% of all lung cancer cases and substantially contributes to cancer deaths worldwide [1]. Both immune checkpoint blockade (ICB) immunotherapy and radiotherapy are first-line treatments for NSCLC. For locally advanced NSCLC, chemoradiation combined with ICB has become the standard treatment. [2–5] However, the curative efficacy of radio-immunotherapy is still suboptimal, and radioresistance remains a significant barrier to improving the antitumor-immunity of IR, limiting the survival benefit of NSCLC from radiotherapy–immunotherapy combinations [6]. Therefore, there is an urgent need to investigate prospective combination modalities to enhance the antitumor immunity of IR.

Radiotherapy can elicit significant immunostimulatory effects, including triggering immunogenic cell death (ICD) and improving the tumor immune microenvironment (TIME) [6]. However, there needs to be more strategies that could effectively boost the antitumor-immunity of radiotherapy in clinical settings due to inadequately elucidated mechanisms. The prevailing view suggests that radiation directly induces DNA damage and micronucleus formation in tumor cells, leading to DNA single- and double-strand breaks (DSBs) [7]. The accumulation of micronuclei and double-stranded DNA (dsDNA) in the cytoplasm leads to the activation of the cytoplasmic DNA receptor cyclic GMP-AMP synthase (cGAS), which activates the downstream STING/type I interferon (I-IFN) signaling pathway and promotes the activation of an antitumor immune response [7]. The generation of DSBs is the central molecular mechanism by which radiation kills cancer cells. It is now well appreciated that DSBs are the central DNA lesion from which immunogenic nucleic acids are created to boost antitumor immunity. [8]

Telomeres protect chromosomes from terminal fusion and maintain genomic stability [9]. Radiosensitivity has been reported to be positively correlated with telomere dysfunction [10, 11]. Most tumors maintain telomere and chromosome stability through telomerase reactivation [11]. Reactivation of telomerase in malignant cells leads to cellular resistance to chemotherapy, radiation, or selective DNA damage response (DDR) inhibitors by avoiding telomere dysfunction-mediated DSBs cascades amplification [8, 12]. Moreover, studies have reported that telomerase promotes the formation of a tumor-suppressive immune microenvironment by strengthening the ability of malignant cells to escape both telomere crisis and immune surveillance [13, 14]. Targeting the pathway of telomere maintenance is a promising strategy for enhancing the immunostimulatory effects of radiotherapy.

Our previous studies have shown that inhibiting telomerase with BIBR1532 significantly enhances cell radiosensitivity by promoting IR-induced telomere dysfunction, while inhibiting the activation of IR-induced ATM/CHK1/CHK2 checkpoint signaling in NSCLC [15]. Additionally, we found PIN2/TERF1-interacting telomerase inhibitor (PinX1), a telomerase binding factor, induces radioresistance through improved telomere stability in esophageal squamous cell carcinoma(ESCC) [16]. PinX1 was initially thought to inhibit the activity of telomerase through its binding to telomerase, which is considered a tumor suppressor [17]. However, many subsequent studies have suggested that high expression of PinX1 contributes to telomere maintenance and is involved in faithful mitotic chromosome segregation, therefore exerting the role of an oncogene. [18, 19] The mechanism by which PinX1 positively regulates telomere maintenance and its role in the antitumor immunity of IR is still unclear, and was further explored in this study.

## Methods

### Cell lines and cell cultures

The human NSCLC cell lines A549 and H460 were cultured in RPMI-1640 medium (Gibco, USA) containing 10% fetal bovine serum (Gibco, USA). Lewis lung cancer (LLC) cells were cultured in DMEM medium (HyClone, USA) with 10% fetal bovine serum. All cell lines were obtained from the Type Culture Center of the Chinese Academy of Sciences (Shanghai, China).

### Transfection with shRNA

PinX1 shRNA and negative control shRNA were incorporated into the lentiviral vector GV112. Following the manufacturer’s protocol, A549, H460 and Lewis lung cancer (LLC) cells were stably transfected with PinX1 shRNA (GeneChem, Shanghai, China) and selected with puromycin(4 μg/ml). All stable cell lines were created with lentivirus transfection. The protocol of cell transfection was described previously [20]. The same method was used to construct A549 cell line with stable knockdown of RBM10. The sequences of shRNA are recorded in Additional file 5: Table. S1.

### In vivo mouse model

All studies were supervised and approved by the Ethics Committee of the First Affiliated Hospital of the University of Science and Technology of China and the Institute. All mice were purchased from SiPeiFu (Beijing, China) and were kept under specific pathogen-free conditions in the Core Facility Center for Medical Sciences of Anhui Provincial Hospital. The ethics number is 2023-N(A)-051.

### Establishment of nude mouse transplantation tumor model

A transplantation tumor model was constructed by injecting 5 × 10^6^ A549 cells subcutaneously into the proximal end of the right hind limb of female BALB/c female nude mouse (4 weeks old). The mice were randomly grouped when the tumors grew to approximately 200 mm^3^ (About 7 days after injection). Specifically, a radiation dose of 10 Gy (2 Gy/times, 1 time/d, 5 consecutive days) was delivered locally to the tumor using a photon beam linear gas pedal (6MV-X-rays) seven days after injection of tumor cells. Tumor size was measured every three days with a vernier caliper, and the tumor volume was calculated using V = (length × width^2^)/2. All mice were sacrificed on day 22.

### Establishment of homozygous mouse models

A total of 5 × 10^6^ LLC cells were injected subcutaneously into the proximal end of the right hind limb of female C57BL/6 mice (4 weeks old). The mice were randomly grouped when the tumor volume was approximately 100 mm^3^ (about 7 days after injection). A radiation dose of 10 Gy was given locally to the tumor (2 Gy /times, 1 time/d, five consecutive days) for radiotherapy. For ICB treatment, mice were intraperitoneally injected with anti-PD-L1 (200 μg/mouse, B7-H1, clone 10 F.9G2, Bio X Cell) every three days starting from exposure to irradiation. For ethical considerations, mice were sacrificed when the tumor volume reached approximately 2 000 mm^3^ or showed signs of morbidity.

### Cell viability assay

Cells were seeded into 96-well plates and, after 24 h, were exposed to a radiation dose of 0 or 6 Gy. Then cell viability was assessed using the Cell Counting Kit-8 (Beyotime, Shanghai, China). Absorbance was measured at 450 nm at the indicated time points.

### Colony formation assay

Cells were seeded in 6-well plates at a density of 100, 200, 400, 800 cells per well for 0, 2, 4, or 6 Gy irradiation, respectively. After adhesion, the cells were irradiated as designed schedule. The treated cells were cultured for 10–14 days when colonies were observed with more than 50 cells. Subsequently, the cells were fixed in ice anhydrous ethanol for 15 min and stained with 0.5% crystal violet for 10 min. Plating efficiency (PE) was defined as the average number of colonies /number of cells seeded. The surviving fraction(SF) was calculated using the following equation: average number of colonies/ (number of cells plated * PE). The SF curve was fitted according to the Linear-quadratic formulation with Graphpad 8.0.

### Western blot

Proteins were extracted from cells on ice using RIPA lysis buffer (Solarbio, Beijing, China) containing protease and phosphatase inhibitors. After 10 min boiling for denaturing, the proteins were separated via sodium dodecyl sulfate–polyacrylamide gel electrophoresis (SDS-PAGE) and then transferred to a 0.45 μm polyvinylidene fluoride membrane (Millipore, Boston, USA). The membrane was blocked with 5% milk for 2 h at room temperature(RT), and subsequently incubated with primary antibodies at 4 ℃ overnight. The next day, the membrane was incubated with HRP-conjugated secondary antibodies at RT for 1 h. Finally, an ECL detection reagent (Millipore) was used to detect the protein signal. Antibody details are shown in Additional file 5: Table S2.

### Immunofluorescence assay

Cells were seeded on polylysine-coated coverslips and then exposed to 6 Gy IR after adherence. After 48 h, the cells were fixed in 4% paraformaldehyde at RT for 15 min, permeabilized with 0.25% Triton X-100 at RT for 10 min, blocked with 5% bovine serum albumin (BSA) at RT for 1 h, and then incubated with primary antibody at 4 ℃ overnight. The next day, the cells were incubated with secondary antibodies (away from light, RT, 1 h). Finally, the cells were washed with PBS 3 times and mounted with anti-fading mounting medium (with DAPI) (Solarbio) to stain the nuclei. Images were acquired with Zeiss LSM800 fluorescence microscope (Jena, Germany). Antibody details are shown in Additional file 5: Table S2.

### PicoGreen staining

Cells were spread on polylysine-coated coverslips and then exposed to 6 Gy radiation. After 48 h of incubation, the cells were placed in a medium containing PicoGreen (Thermo Fisher, USA) (3 μl / ml) and incubated at 37 °C for 1 h. The cells were washed, fixed, and then covered with an anti-fluorescence quenching DAPI-containing capping agent (Solaibo) for confocal microscopy observation.

### Flow cytometry analysis

The detection of apoptosis and the cell cycle was performed as described in previous literature [15]. For the detection of TME, the peripheral blood (PB), tumor, and spleen of mice were made into single-cell suspensions. After red blood cell lysis (Biolegend), cells were counted and, at a number of 3–5 × 10^6^ per tube were incubated with fluorescently labeled antibody cocktail (30 min, 4℃, in the dark). For cell surface staining, the single-cell suspensions were incubated with the antibodies on ice for 30 min. For intracellular staining, cells were fixed and then permeabilized with intracellular staining/permeabilization solution (Bio Legend, San Diego, CA, USA) and then stained with intracellular antibodies on ice for 30 min. Data were collected using an LSRFortessa flow cytometer (BD Bioscience) and analyzed with FlowJo 10 software. Details of the antibodies are shown in Additional file 5: Table S2.

### Co-immunoprecipitation (Co-IP) and Mass spectrometry (MS)

Cells were lysed on ice with IP lysis buffer (Beyotime). Lysates were immunoprecipitated with antibodies at 4 °C overnight. The immunocomplexes were captured using Protein A/G Magnetic Beads (MedChemExpress, Shanghai, China) and washed with IP lysis buffer. The eluate was separated by SDS-PAGE and stained with a Fast Silver Stain Kit (Beyotime). MS was performed using Wininnovate Biotechnology. Western blotting was used to detect the protein of interest in the co-IP product. The antibodies used in these assays are shown in Additional file 5: Table S2.

### Statistic snalysis

All statistical analyses were conducted with the GraphPad Prism 8.0.2 software (GraphPad, Software). Data were generated from at least three repeats of different biological experiments and expressed as mean ± SD. An unpaired t test was applied for comparison of means of two groups with normal (or about normal) distributions. To compare means between > 2 groups, we used one-way ANOVA with multiple comparison corrections (Dunnett’s test). Survival curves were plotted using the Kaplan- Meier method and compared using a log-rank test. A value of *p* < 0.05 was considered as statistical significance.

## Results

### Silencing PinX1 enhances NSCLC radiosensitivity in vitro and in vivo

**Fig. 1:**
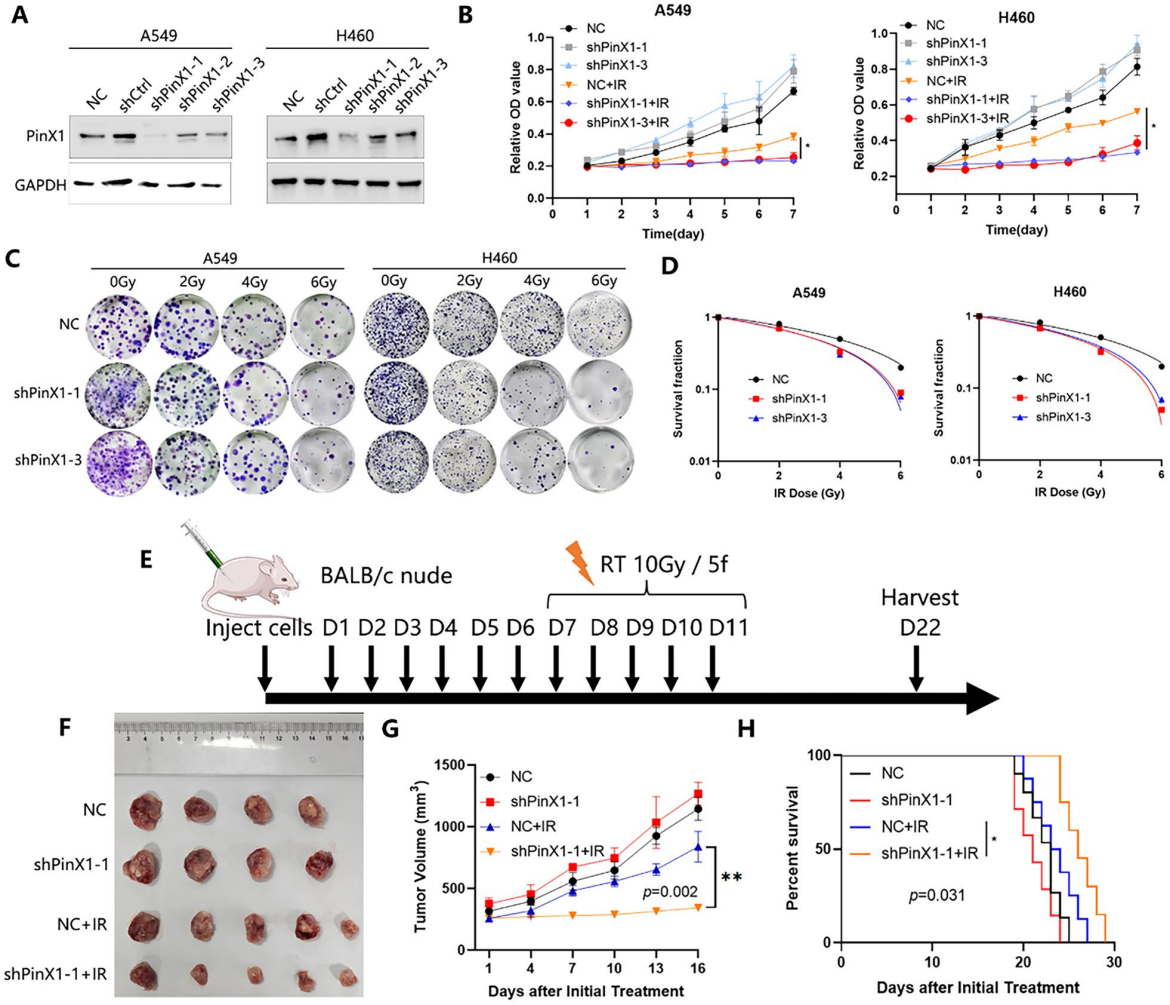
Silencing PinX1 enhances NSCLC radiosensitivity in vitro and in vivo. **A** A549 and H460 cells were transfected with a control or shPinX1 lentivirus and analyzed by western blotting. **B** Cell viability was analyzed via Cell Counting Kit-8 (CCK-8) assays. **C** Representative images of colony formation of cells irradiated with 0, 2, 4, and 6 Gy. **D** The cell survival curves were fitted with the linear-quadratic model. **E** When the tumor grew to a volume of approximately 200 mm3, mice were randomly divided into the following four groups: NC (negative control), shPinX1, IR, and IR plus shPinX1 (n = 5 mice/group). Experimental schedule of shPinX1 and IR combination treatment for A549 tumor-bearing nude mice. **F**. Representative photos of tumor xenografts 14 days after the initiation of IR. **G** Xenograft tumor volumes are shown in the graphs. Two-way ANOVA with Tukey’s multiple comparison test was conducted for tumor volume analysis with GraphPad Prism 8.0.2. Data are presented as the mean ± standard deviation. **H** Survival data were compared using the log-rank Mantel-Cox test.(n = 8 mice/group)**.** *: *p* < 0.05, **: *p* < 0.01, ***p: < 0.001, ****: *p* < 0.0001

To investigate the effect of PinX1 on radiosensitivity in NSCLC, cell lines were constructed with stable knockdown of PinX1 in A549 and H460 cell lines. Western blot analysis verified the knockdown efficiency (Fig. 1A). The proliferation activity of cells treated with 6 Gy IR was assessed using CCK-8 assay. The results showed that silencing PinX1 combined with IR significantly inhibited the cell viability of NSCLC (Fig. 1B). Silencing PinX1 combined with IR significantly inhibited the clone formation of NSCLC cells compared with only IR (Fig. 1C, D). Then, the effect of PinX1 on radiosensitivity was investigated in vivo. The experimental protocol is illustrated in Fig. 1E. In parallel with the in vitro experiments, the in vivo assays showed that silencing PinX1 plus IR significantly delayed tumor growth (mean tumor volume ± SEM: 838.55 ± 123.90 mm^3^ IR vs 343.08 ± 13.20 mm^3^ silencing PinX1 + IR, *p* = 0.002; Fig. 1F, G) and improved survival(8.56 ± 0.85 days IR vs 11.13 ± 0.74 days silencing PinX1 plus IR, *p* = 0.031; Fig. 1H) in nude mice compared with radiation alone. On the other hand, silencing PinX1 enhanced IR-induced DNA DSBs (Additional file 1: Fig S1A–C) and cell apoptosis (Additional file 1**: **Fig. S1D, E).

### Silencing PinX1 enhanced IR-induced telomere dysfunction and boosted the activation of cGAS-STING pathway

**Fig. 2:**
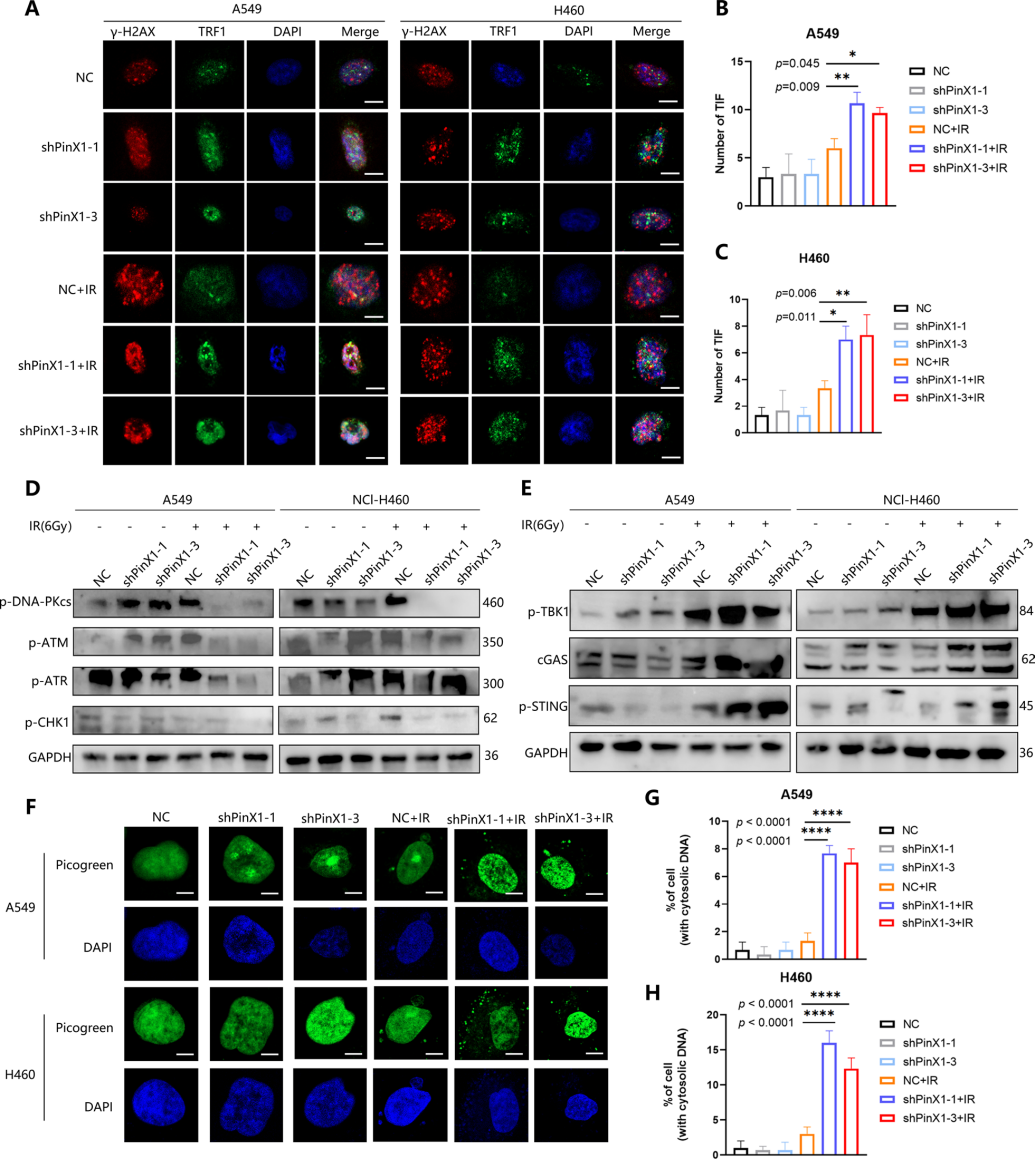
Silencing PinX1 enhanced IR-induced telomere dysfunction and boosted the activation of cGAS-STING pathway. **A**–**C** Silencing PinX1 increased the percentage of telomere dysfunction-induced foci (TIF)-positive cells after IR exposure. Cells were double stained with an anti-TRF1 antibody for telomere markers (green) and an anti-phosphorylated histone H2A.X (Ser-139) antibody for γ-H2AX foci (red). Nuclei were counterstained with DAPI (blue). Merged images show DNA damage at telomeres. Arrows indicate double-stained areas (yellow). Scale bar, 10 μm; n = 3/group. **D** Expression of phosphorylated ATM, ATR, DNA-PKcs, and CHK1 was detected with Western blot at the indicated times after 6 Gy IR. **E** Expression of p-TBK1, p-STING and cGAS was detected with western blot at the indicated times after 6 Gy IR. **F**–**H** Representative images and quantitative analysis of PicoGreen staining. Cells with cytosolic DNA were counted. Scale bar, 10 μm. n = 3/group. *: *p* < 0.05, **: *p* < 0.01, ***p: < 0.001, ****: *p* < 0.0001

We next investigated whether PinX1 could influence IR-induced telomere dysfunction in NSCLC cells (Calculations in telomere dysfunction-induced foci (TIF), where TRF1 and γ-H2AX co-localize, represent telomeric DNA damage). The incidence of telomere dysfunction judged by TIF was markedly increased in PinX1-silenced cells compared with control cells after IR exposure (Fig. 2A–C).

Ataxia telangiectasia-mutated (ATM) is a core kinase responsible for the cellular response to IR-induced DSBs [15]. Western blot showed that silencing PinX1 significantly inhibited radiation-induced ATM/checkpoint kinase 1(CHK1) phosphorylation, and decreased phosphorylated recombinant antitrypsin related protein(ATR) and DNA-dependent protein kinase catalytic subunit (DNA-PKcs) suggesting a significant inhibition of DNA DSBs repair (Fig. 2D).

Previous studies have shown that RT can disrupt the DNA structure of tumor cells, resulting in a large number of DNA fragments in their cytoplasm, which activates the cGAS-STING pathway, thereby causing the release of IFN-I and inducing a stronger anti-tumor immune response [21–24]. It was observed that three critical proteins, cGAS, phosphorylated TANK-binding kinase 1 (TBK1) and stimulator of interferon genes (STING), were significantly increased in shPinX1 + IR group at the expression level (Fig. 2E). Defectively, DSB repair leads to the accumulation of damaged DNA, which leaks into the cytoplasm and triggers the cytoplasmic DNA receptor cGAS, which in turn activates STING and its downstream proteins. Picogreen is a double-stranded DNA-specific fluorescent dye that binds to DNA molecules and emits a fluorescent signal. As expected, silencing PinX1 induced the accumulation of cytoplasmic dsDNA upon IR exposure (Fig. 2F–H).

### Silencing PinX1 combined with IR enhanced the immune microenvironment

**Fig. 3:**
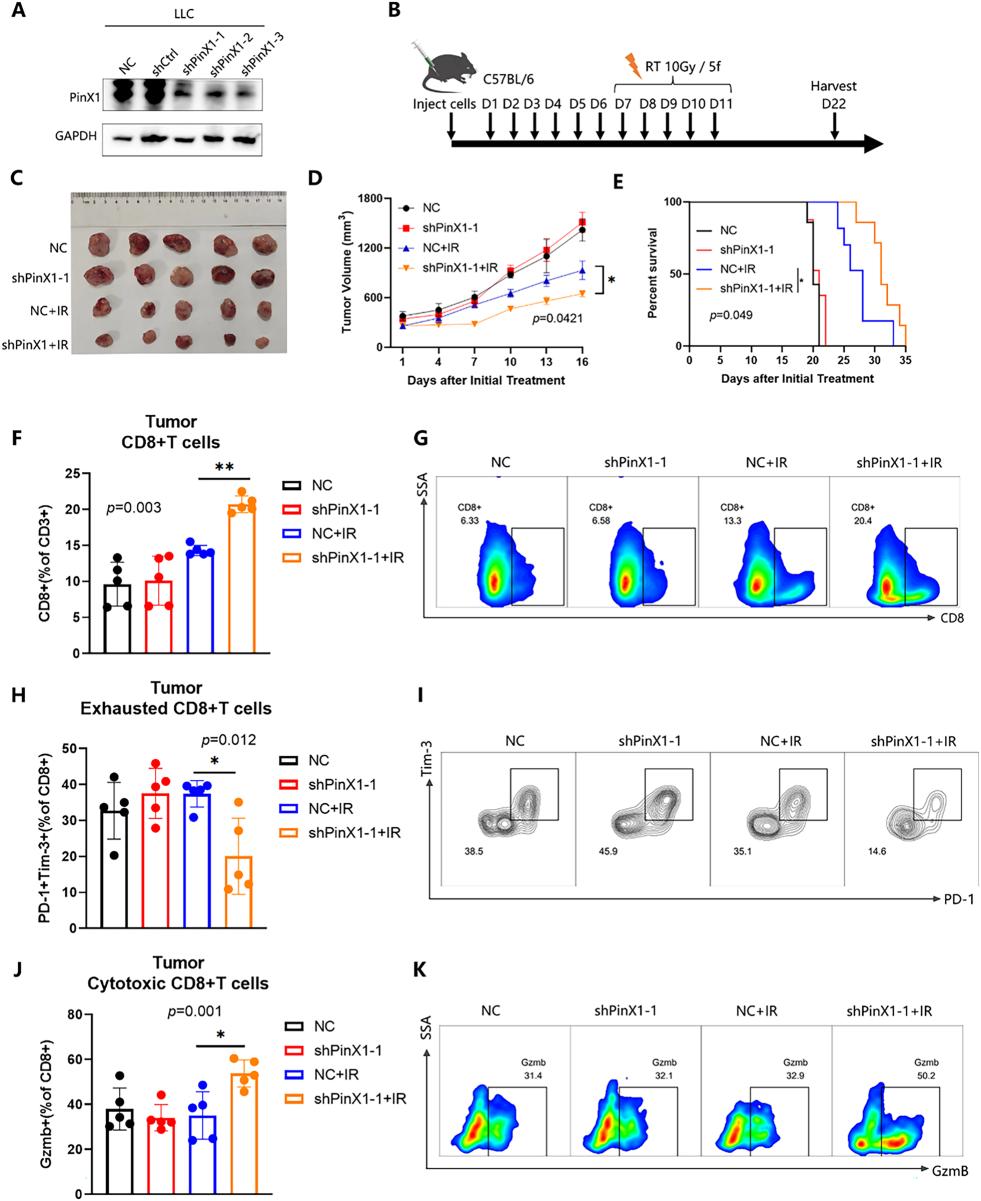
Silencing PinX1 combined with IR increased CD8^+^ T cell recruitment. **A** Lewis cells were transfected with a control or shPinX1 lentivirus and analyzed by western blotting. B. Experimental schedule of varied treatment regimens for LLC tumor-bearing mice. **C** Representative photos of tumor xenografts 14 days after the initiation of IR. **D** Tumor volume of xenografts was shown in the graphs. Two-way ANOVA with Tukey’s multiple comparison test was conducted for tumor volume analysis with GraphPad Prism 8.0.2. n = 5 mice/group. **E** Survival data were compared using log-rank Mantel-Cox test. n = 8 mice/group. **F**–**K** Representative flow cytometry staining and frequencies of total CD8^+^/CD3^+^ cells, PD-1^+^ Tim-3^+^/CD8^+^ T cells, and Gzmb^+^ /CD8^+^ T cells in tumors. n = 5 mice/group. Data are presented as the mean ± standard deviation, *: *p* < 0.05, **: *p* < 0.01, ***p: < 0.001, ****: *p* < 0.0001

cGAS-STING pathway is a key pathway linking radiotherapy and immunity [25]. Based on the above results, silencing PinX1 may play a role in regulating the immunostimulatory effects of IR. To verify this hypothesis, we constructed LLC cells with stable PinX1 knockdown (Fig. 3A). Next, we performed the following treatments on LLC-homozygous C57/BAL6 mice (Fig. 3B). We found that silencing PinX1 combined with IR significantly inhibited tumor growth (Fig. 3C, D) mean tumor volume on day 16 ± SEM: 930.2 ± 107.2 mm^3^ IR vs. 649.1 ± 44.3 mm^3^ shPinX1 plus IR, *p* = 0.0421) and dramatically improved the survival of the homograft mouse(*p* = 0.049) (Fig. 3E).

TIME consists of the interaction of different immune cells in the TME and plays an important role in tumorigenesis, progression and therapeutic response [26, 27]. Many studies have reported that RT can remodel TIME to attenuate the immunosuppressive state, exert antitumor effects, and exhibit enhanced immune response and therapeutic efficacy when used in combination with immunotherapy [28, 29]. For TIME, silencing PinX1 combined with IR significantly increased the percentage of CD8^+^ T cells (14.3 ± 0.71 IR vs 20.72 ± 1.16 shPinX1 + IR, % of CD3^+^ cells, *p* = 0.003, Fig. 3F, G) and Granzyme B^+^(GzmB) cytotoxic CD8^+^ T cells (35.12 IR ± 10.54 vs 53.80 ± 6.06 shPinX1 + IR, *p* = 0.001, Fig. 3J, K) compared to the IR group, as well as markedly decreased the percentage of CD8^+^ PD-1^+^ Tim3^+^ exhausted CD8^+^T cells in tumor tissues(37.40 ± 3.69 IR vs 20.08 ± 10.58 shPinX1 + IR, *p* = 0.012, Fig. 3H, I). Collectively, these results suggested that silencing PinX1 enhanced the antitumor immunity of IR. In addition, we found that silencing PinX1 combined with IR significantly increased CD8^+^ T infiltration in PB(*p* = 0.006, Additional file 2: Fig. S2A) and spleen (*p* = 0.0019, Additional file 2: Fig. S2D). Interestingly, the proportions of cytotoxic CD8^+^T (*p* = 0.0477, Additional file 2: Fig. S2B) as well as central memory/effector CD8^+^T cells (Additional file 2: Fig. S2C) in PB were also significantly elevated in shPinX1 + IR group, suggesting the potential of targeting PinX1 in combination with IR to improve the peripheral immune microenvironment.

### Silencing PinX1 potentiates the antitumor efficacy of radioimmunotherapy

**Fig. 4:**
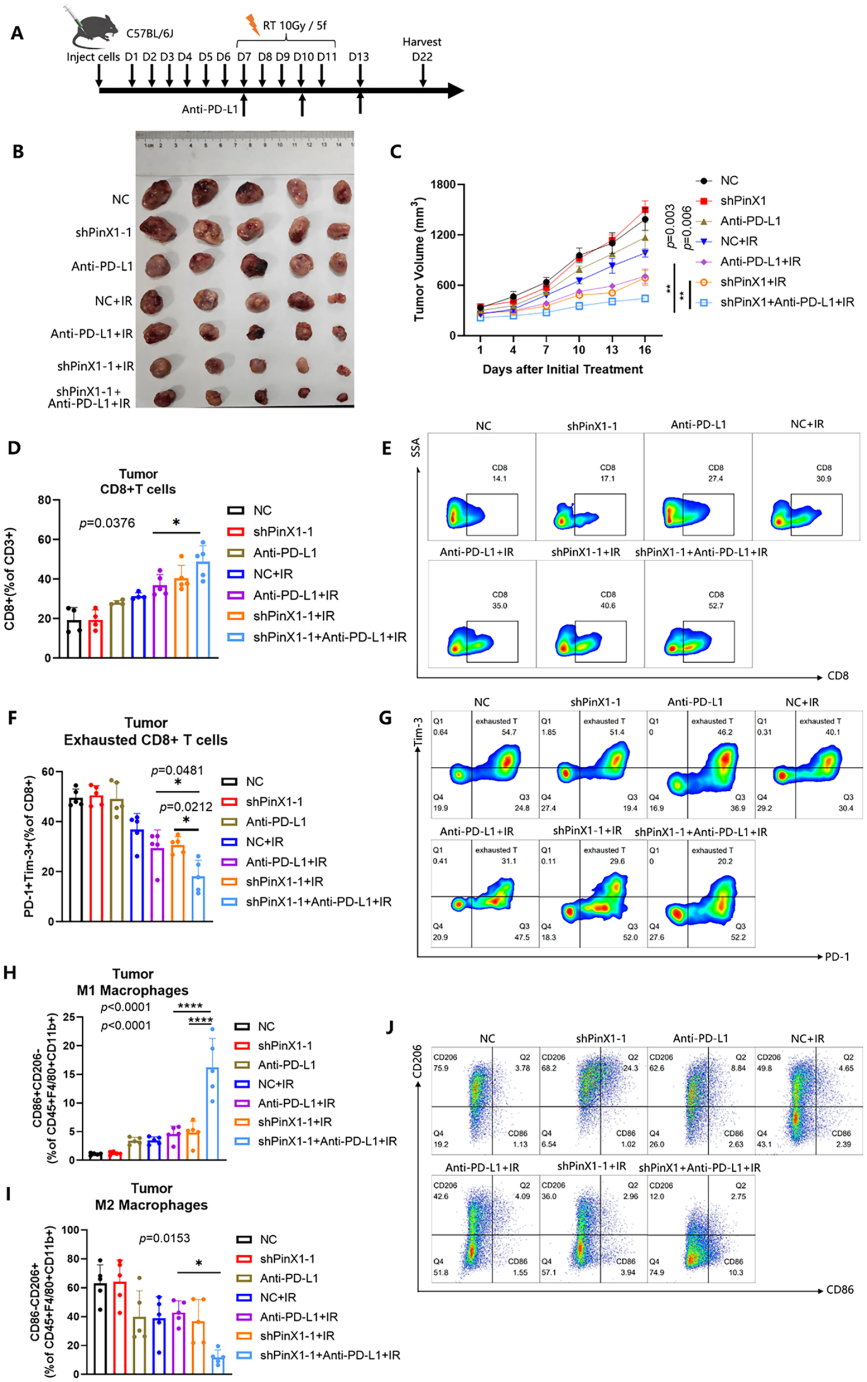
Silencing PinX1 potentiates the antitumor efficacy of radioimmunotherapy. **A** Experimental schedule of varied treatment regimens for LLC tumor-bearing mice. **B** Representative photos of tumor xenografts 14 days after the initiation of IR. **C** Tumor volume of xenografts was shown in the graphs. Two-way ANOVA with Tukey’s multiple comparison was conducted for tumor volume analysis with GraphPad Prism 8.0.2. n = 5 mice/group. **D**, **E** Representative flow cytometry staining and frequencies of total CD8^+^/CD3^+^ cells in tumors. n = 5 mice/group. **F**, **G** Representative flow cytometry staining and frequencies of PD-1^+^ Tim-3^+^/CD8^+^ T cells in tumors. n = 5 mice/group. **H**–**J**. Representative flow cytometry staining and frequencies of CD86^+^CD206^−^/CD45^+^F4/80^+^CD11b^+^ cells and CD86^−^CD206^+^/CD45^+^F4/80^+^CD11b^+^ cells cells in tumors. n = 5 mice/group. Data are presented as the mean ± standard deviation. *: *p* < 0.05, **: *p* < 0.01, ***:*p* < 0.001, ****:*p* < 0.0001

Based on the PACIFIC study, concurrent chemoradiotherapy combined with durvalumab is the first-line treatment for locally advanced unresectable stage III NSCLC patients. To determine whether PinX1 affects the efficacy of radioimmunotherapy, several treatment regimens were performed on LLC-bearing C57BL/6 mice (Fig. 4A). For RT, a radiation dose of 10 Gy was given locally to the tumor (2 Gy /times, 1 time/d, five consecutive days) when the tumor volume was approximately 100 mm^3^ (about 7 days after injection). For ICB treatment, mice were intraperitoneally injected with anti-PD-L1 (200 μg/mouse) every three days when the tumor volume reaches approximately an average of 100 mm^3^. Radioimmunotherapy means a combination of RT and ICB treatment. Results showed that tumor growth in the shPinX1 + Anti-PD-L1 + IR group showed significant delay compared to that in the Anti-PD-L1 + IR group (*p* = 0.003) and shPinX1 + IR group (*p* = 0.006) (Fig. 4B, C).

Next, we explored the effects of the above treatments on the TIME. Compared to radioimmunotherapy alone, silencing PinX1 combined with radioimmunotherapy significantly increased the infiltration of CD8^+^ T cells in tumor tissues (*p* = 0.0376) (Fig. 4D, E). In addition, the percentage of exhausted CD8^+^T cells in the shPinX1 combined with radioimmunotherapy group was much lower in compared with the IR + anti-PD-L1 group(*p* = 0.0481) or IR + shPinX1 group(*p* = 0.0212) in tumor tissues(Fig. 4F, G). Moreover, we detected that in tumor tissues, the proportion of M1-like macrophages was significantly increased in the shPinX1 + Anti-PD-L1 + IR group compared to either the Anti-PD-L1 + IR group (*p* < 0.0001) or the shPinX1 + IR group (*p* < 0.0001), whereas the proportion of M2-like macrophages was markedly decreased in the shPinX1 + Anti-PD-L1 + IR group compared to the Anti-PD-L1 + IR group (*p* = 0.0153), which suggests that shPinX1 combined with radioimmunotherapy may be involved in the reprogramming of macrophages from M2-type to M1-type (Fig. 4H–J). Interestingly, shPinX1 conbined with radioimmunotherapy group decreased the percentage of regulatory T cells and increased the percentage of IFN-γ^+^ CD8^+^ T cells in spleen tissues, and increased the percentage of dendritic cells (DCs) in PB (Additional file 3: Fig. S3A–E). These results suggested that silencing PinX1 combined with IR significantly improved the TIME and contributed to antitumor responses of radioimmunotherapy.

### Interaction of PinX1 with RBM10 promotes telomerase transport to telomeres

**Fig. 5:**
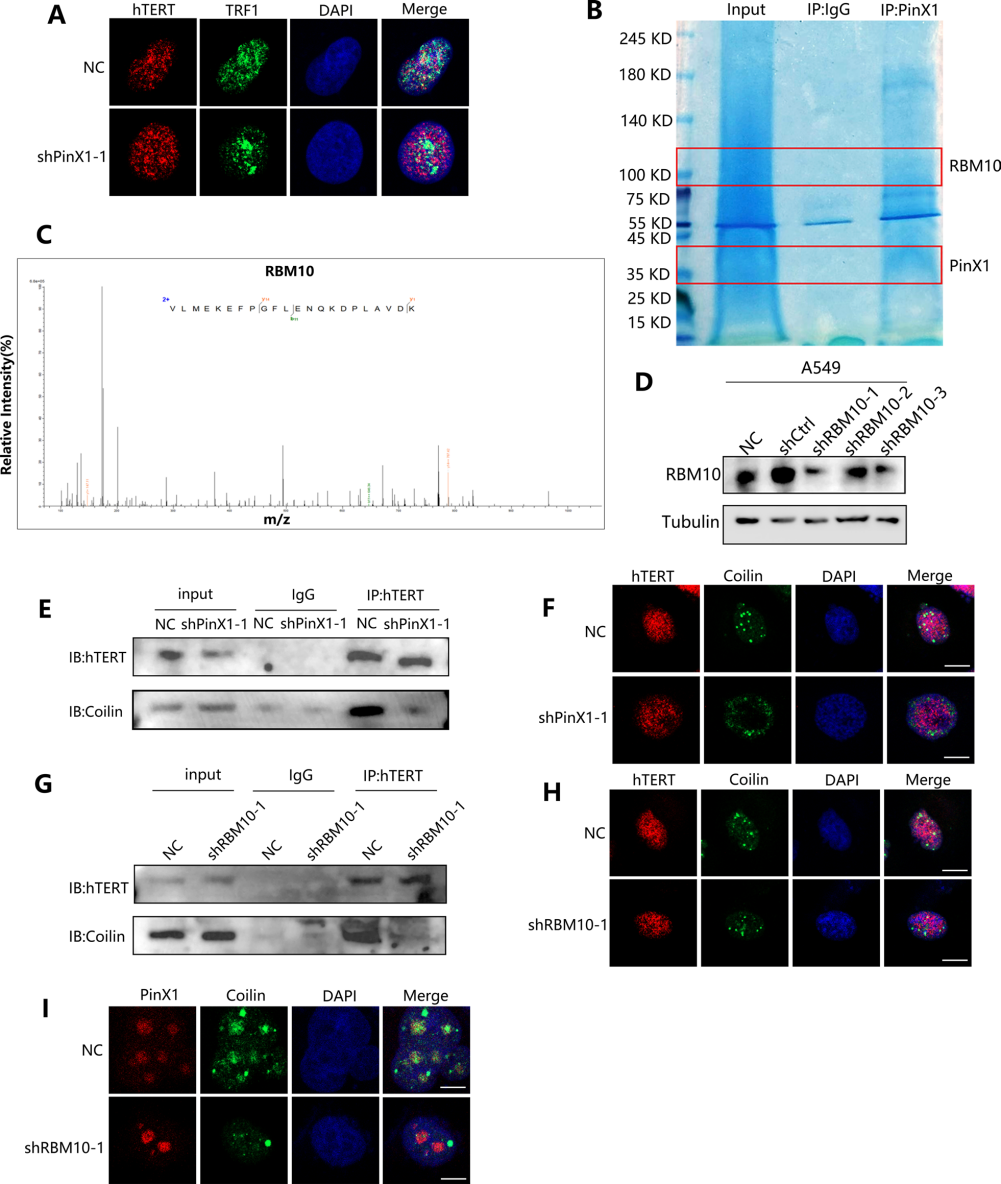
Interaction of PinX1 with RBM10 promotes telomerase transport to telomeres. **A** Knockdown of PinX1 reduced hTERT co-localization with TRF1. **B** Coomassie brilliant blue staining image of IP assay using anti-PinX1 antibody and IgG in A549 cells. **C** Mass spectrometry image of RBM10. **D** A549 cell lines were transfected with a control or shRBM10 lentivirus and validated by western blotting. **E**–**F** IP assay and immunofluorescence to detect the content of hTERT bound to coilin in shNC and shPinX1-1 A549 cells. **G**, **H** IP assay and immunofluorescence to detect the content of hTERT bound to coilin in shNC and shRBM10-1 A549 cells. **I**. Knockdown of RBM10 reduced PinX1 co-localization with coilin

PinX1 has been reported to play a role in the translocation of telomerase to telomeres, which is supported by our demonstration in the present study that PinX1 silencing resulted in a significant reduction in the binding of TRF1, a telomere sheltering protein, to telomerase (Fig. 5A). Related studies have found that telomere dysfunction due to, for example, mislocalization of telomerase, enhances cellular sensitivity to DNA damage reagents and radiation [10, 12]. To explore the mechanism by which silencing PinX1 enhances IR-induced antitumor immunity, we identified the potential PinX1-binding protein RBM10 by IP-MS analysis (Fig. 5B, C and Additional file 6) and further verified the interactions by co-IP and immunofluorescence (Additional file 4: Fig. S4A, B). RNA-binding motif protein 10 (RBM10) encodes an RNA-binding protein involved in the regulation of splicing, and is mutated at a high frequency in a variety of tumors, especially lung adenocarcinoma [30]. Coilin is the major protein component of Cajal bodies (CBs), nuclear suborganisms that serve as maturation and assembly sites for the splicing machinery [31]. Moreover, CBs are thought to mediate the transport of telomerase to telomeres [32, 33]. Our study shows that RBM10 co-localizes with coilin. (Additional file 4: Fig. S4C) We therefore hypothesized that the interaction of PinX1 and RBM10 is likely to be involved in the regulation of telomerase recruitment, thereby maintaining telomere stability.

To verify this hypothesis, we constructed cell lines with stable knockdown of RBM10 in A549 cell line and verified the knockdown efficiency by western blot.(Fig. 5D) We found that after silencing PinX1 or RBM10 to disrupt their interaction, the co-localization and interaction between hTERT and coilin were significantly reduced(Fig. 5E–H). In addition, silencing RBM10 similarly significantly reduced PinX1 co-localization with coilin, which indicated that PinX1 requires RBM10 assistance to reach CBs (Fig. 5I). These results suggest that PinX1 may be involved in CBs-mediated translocation of telomerase to telomeres through interaction with RBM10, thereby enhancing telomere maintenance and subsequently leading to radioresistance.

## Discussion

Our study indicated that silencing PinX1 enhances radiosensitivity and antitumor-immunity of RT by enhancing IR-induced telomere dysfunction, therefore improving the antitumor efficiency of radioimmunotherapy.

PinX1 was initially identified as a telomerase-binding protein with a highly potent inhibitor of telomerase activity [17]. However, many studies have suggested that PinX1 positively regulates telomere maintenance and promotes genome stability in tumor cells [18, 34]. The role of PinX1 in tumorigenesis and progression varies widely among cancer types [16, 35, 36]. For NSCLC, previous studies have shown that PinX1 inhibits tumor cell proliferation [37, 38]. Our previous studies have shown that the high expression of PinX1 induced radioresistance through promoting telomere maintenance and chromosome stabilization, and targeting PinX1 significantly enhances radiosensitivity via increasing IR-induced telomere dysfunction in ESCC [16]. In the in vitro experimental part of our study (including colony formation assay and CCK-8 assay), knockdown of PinX1 also increased cell proliferation of tumor cells to a certain extent, however, when combined with RT, cell proliferation appeared significantly downregulated relative to RT alone. In the in vivo part of the experiment, our results showed that the tumor volume did not significantly increase in the shPinX1 group compared with the Negative Control group, and only when shPinX1 was combined with RT, the tumor volume was significantly reduced. We considered that it was due to the fact that the PinX1 shRNA sequence used in our in vivo experiments was different from that reported in the literature.

Previous researches have demonstrated the mechanism by which IR killing tumor cells could be directly damaging their DNA [39]. There is now a growing recognition that RT can act as an "in situ vaccine" to activate host anti-tumor immunity, which cause tumor cells to ICD, as well as optimize TIME by regulating the expression of major histocompatibility complex-I (MHC-I) and intercellular adhesion molecules (I-CAMS) in vascular endothelial cells [40, 41]. Accordingly, the combination of RT and immunotherapy has been developed. However, radioresistance and immune tolerance limit the efficacy of radioimmunotherapy, and tumor progression still occurs in roughly 2/3 of NSCLC patients, thus a safe and effective strategy for enhancing radiosensitivity and improving the immunostimulatory effects of IR is urgently needed [2, 4]. Telomere dysfunction can interfere with the properness of radiation-induced DSBs repair, thus giving rise to large amounts of DNA damage, and these free DNA fragments are captured by DCs and activate STING-dependent type I interferon activation, leading to radioresistance and immunoresistance [42, 43]. It is now well appreciated that misplaced DNA generated from DSBs or faulty DDR can induce antitumor immunity through activating primitive pathogen pattern recognition receptors in malignant cells. [8, 44] These results suggested that targeting telomerase/telomeres is promising to boost the antitumor immunity of RT. Herein, in this study, we demonstrated that silencing PinX1 destroyed the function of telomerase in telomere maintainance in vitro and in vivo, thus effectively promoting the immune-activating effects of IR and significantly enhancing the antitumor efficacy of radioimmunotherapy in NSCLC cells.

Our study found that silencing PinX1 combined with IR significantly increased CD8^+^ T cells infiltration as well as CD8^+^ cytotoxic T lymphocytes in tumor tissues and peripheral immune organs. The infiltration and activation of CD8^+^ T cells have a strong correlation with the prognosis of several types of cancer [45–47]. Additionally, CD8^+^ cytotoxic T cells-mediated antitumor immunity has been suggested as a determinant of ICB effectiveness. Radiation promotes CD8^+^T cells infiltration by inducing DSBs to activate the cGAS-STING pathway and enhances anti-tumor immune responses by releasing tumor-associated antigens and chemokines [44]. Indeed, silencing PinX1 markedly enhanced the activation of the cGAS-STING pathway by increasing IR-induced free dsDNA production.

In normal conditions, CD8^+^ T cells kill tumor cells and suppress tumor proliferation [47]. However, these T cells gradually lose their immune effector function, which is termed "exhausted CD8^+^ T cells" (Tex) [48]. Tex can inhibit T cells from exerting anti-tumor immunity by expressing immunosuppressive receptors such as PD-1 and T cell immunoglobulin mucin(Tim-3) [49, 50]. When T cells are exhausted, tumor cells can evade the immune system and grow uncontrollably. Therefore, preventing CD8^+^ T cells from exhaustion is one of the main goals of tumor immunotherapy. However, the mechanism of the production of Tex is inadequately elucidated [51]. In our study, silencing PinX1 in combination with radioimmunotherapy significantly reduced the proportion of Tex in tumor tissues. This finding suggested some roles of telomere dysfunction in inhibiting T cells exhausted.

We also found that silencing PinX1 combined with radioimmunotherapy dramatically promoted M1 macrophage polarization in NSCLC. We know that in lung cancer, macrophages are a major component of the TIME [52]. There are two main phenotypes of macrophages, pro-inflammatory (M1-type) macrophages and anti-inflammatory (M2-type) macrophages [53]. M1-type macrophages can kill tumor cells through mediating cytotoxicity directly or mediating antibody-dependent cytotoxicity, while pro-tumourigenic M2-type macrophages is committed to tumor growth, invasion and metastasis [53]. Therefore, reprogramming of tumor-associated macrophages from M2-type to M1-type is critical for alleviating the immunosuppressive state of the TIME and controlling tumor growth. Other than induced ICD, optimized radiation can also promote the conversion of M2 macrophages to M1 in the TIME [54]. These findings may explain why targeting PinX1 could improve the tumor-suppressive immune microenvironment.

**Fig. 6:**
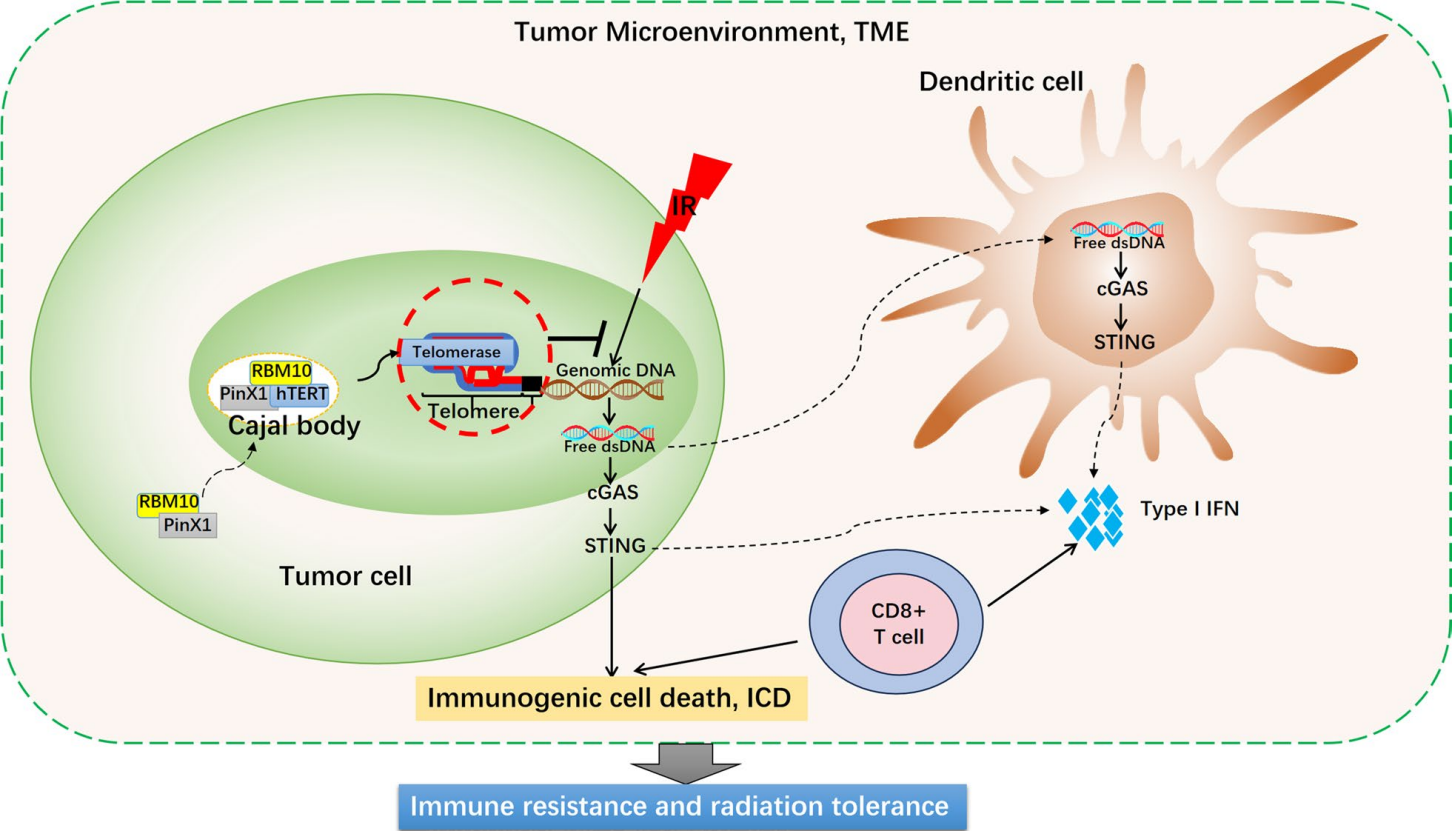
Schematic diagram for the potential mechanisms by which PinX1/RBM10 interaction mediates telomerase transport to telomeres and thus regulates radiosensitivity in NSCLC. Telomere dysfunction can interfere with the properness of radiation-induced DSBs repair, resulting in extensive DNA damage. These free dsDNA fragments are trapped by DCs and activate STING-dependent activation of type I interferons, leading to radiosensitisation and immune activation. PinX1 interacts with RBM10 and assists in the localisation of telomerase to the Cajal body. Subsequently, telomerase continues to recruit to telomeres, maintains telomere stability, and inhibits activation of the cGAS-STING pathway and its mediated IR-induced anti-tumour immune effects, leading to radioresistance and immune tolerance in NSCLC

Most malignant cancer cells compensate for the replicative shortening of telomeres through telomerase reactivation to achieve immortality [9]. Related studies have also shown that telomerase inhibits DNA damage in malignant tumor cells by maintaining telomere stability, which may promote the formation of a tumor-suppressive immune microenvironment [10]. Therefore, telomerase activation is a guarantee for most malignant tumors to maintain genomic stability, and it is also a critical factor affecting radiosensitivity and the antitumor immune activation effect of RT [14–16]. In this study, we revealed that PinX1 knockdown inhibited telomerase binding to telomeres. Furthermore, we identified a new protein interacted with PinX1, RBM10. RBM10 is a component of the spliceosome and negatively regulates the splicing of target exons by binding to specific regions in pre-mRNAs [30]. Notably, RBM10 regulates hTERT gene splicing and inhibits pancreatic cancer progression [55, 56]. In addition, we found that the Cajal body’s marker protein, coilin, co-localized with RBM10/PinX1. Related studies have shown that recruitment of telomerase to CBs is a critical step in the transport of telomerase to telomeres, suggesting that the PinX1/RBM10 interaction may be involved in the regulation of telomerase transport to telomeres. Using co-IP and immunofluorescence, we demonstrated that after the knockdown of PinX1/RBM10, the interaction and co-location between hTERT and CBs or telomeres were both significantly reduced, which validates the positive regulatory effect of PinX1/RBM10 interaction on telomerase transporting to telomeres. The localization of telomerase to CBs is a key step in its recruitment to telomeres, which is a prerequisite for telomerase to play its role in maintaining telomere stability [57]. Genomic instability caused by telomere dyfunction attenuates the repair process of IR-induced DNA DSBs [12]. Mender et al. have found that inhibiting telomerase can enhance anti-tumor immunity by triggering the STING/IFN-I pathway of cytoplasmic DNA perception in DCs [42]. Therefore, we hypothesised that the interaction of PinX1 with RBM10 mediates telomerase localisation to CBs and promotes telomerase recruitment to telomeres, thereby maintaining telomere maintenance and mediating radioresistance and immunosuppression in NSCLC. Figure 6 depicts this possible mechanism.

As a strong inhibitor of telomerase activity, PinX1 must leave telomerase when assisting in its transport to telomeres in order for telomerase to exert its role in prolonging telomeres. Our further study showed that the interaction between PinX1 and hTERT mainly occurs in the non-S phase of the cell cycle, whereas in the S phase, when telomerase extends telomeres, the binding of hTERT to TPP1 significantly inhibits the interaction between hTERT and PinX1 (Additional file 4: Fig. S4D, E). Recent studies have showed that TPP1 plays a central role in telomerase recruitment [58–60]. Our study suggested that PinX1 and TPP1 may synergistically regulate the transport and recruitment of telomerase to telomeres in different cell cycles, which is worthy of further study.

Our studies had several limitations. Our researches were based on animal models and cell experiments. More clinical samples should be collected for translational investigation. In addition, transgenic mice would be an ideal model for further validation of PinX1 function. Moreover, more anti-tumor immune effects need to be investigated, such as abscopal effects and antigen presentation.

Our study demonstrated the critical role of PinX1 in modulating radiosensitivity in NSCLC for the first time. Moreover, targeting PinX1 enhanced radiotherapy-induced anti-tumor immunity. Notably, cells deficient in PinX1 benefit from a combination treatment with RT or radioimmunotherapy, suggesting a potential role for targeting telomeres/telomerase in radioimmunotherapy, which may help the development of individualized strategies for the treatment of NSCLC (Additional file 7).

## Conclusions

In conclusion, our research found that PinX1 mediates radioresistance in NSCLC by assisting telomerase transport to telomeres through interactions with RBM10. Moreover, down-regulation of PinX1 combined with RT could effectively enhance anti-tumor immunity. Our work indicated that targeting PinX1 may have a promising potential for NSCLC treatment. Notably, this study presents a theoretical basis for fu human research on the NSCLC treatment.

## Supplementary Information

**Additional file 1: Fig S1.** Silencing of PinX1 enhances IR-induced DNA damage and cell apoptosis. **A**–**C** Immunofluorescence was performed to detect γ-H2AX foci formation. Scale bar: 10 μm. n = 3/group. **D**, **E** Knockdown of PinX1 enhances IR-induced apoptosis. Forty-eight hours after radiation therapy, cell apoptotic death events were monitored with Annexin V/PI staining and flow cytometry assays. n = 3/group. Data are expressed as the mean ± standard deviation. *: *p* < 0.05, **: *p* < 0.01, ***p: < 0.001, ****: *p* < 0.0001.

**Additional file 2: Fig. S2. **Silencing PinX1 improves the tumor microenvironment in peripheral immune organs. **A**, **B** Representative flow cytometry staining and frequencies of total CD8^+^/CD3^+^ cells in PB. n = 5 mice/group. **C**, **D** Representative flow cytometry staining and frequencies of GzmB^+^/CD8^+^ T cells in PB. n = 5 mice/group. **E**–**G** Representative flow cytometry staining and frequencies of, CD44^+^CD62L^−^/CD8^+^ and CD44^−^CD62L^+^/CD8^+^ T cells in PB. n = 5 mice/group. **H**, **I** Representative flow cytometry staining and frequencies of total CD8^+^/CD3^+^ cells in the spleen. n = 5 mice/group. Data are presented as the mean ± standard deviation. *: *p* < 0.05, **: *p* < 0.01, ***: *p* < 0.001.

**Additional file 3: Fig. S3. **Silencing PinX1 further enhances the immune activation of peripheral immune organs on radioimmunotherapy. **A**, **B** Representative flow cytometry staining and frequencies of MHC II^+^CD11c^+^/live dendritic cells in PB. n = 5 mice/group. **C**, **D** Representative flow cytometry staining and frequencies of CD25^+^Foxp3^+^/CD4^+^ T cells in the spleen. n = 5 mice/group. **E**, **F** Representative flow cytometry staining and frequencies of IFN-γ^+^/CD8^+^ T cells in the spleen. n = 5 mice/group. Data are presented as the mean ± standard deviation, *: *p* < 0.05, **: *p* < 0.01, ***p: < 0.001, ****: *p* < 0.0001.

**Additional file 4: Fig. S4. **PinX1 and TPP1 may synergistically regulate the transport of telomerase to telomeres in different cell cycles. **A**, **B** IP and immunofluorescence demonstrated the interaction between PinX1 and RBM10 in A549 cells. Scale bar: 10 μm. **C** Immunofluorescence proved the co-localization of colin and RBM10 in A549 cells. Scale bar: 10 μm. **D** After enrichment of S-phase cells by thymidine double blocking, the enrichment efficiency was verified using flow cytometry in A549 cells. **E** Knockdown of PinX1 enhanced the interaction of TPP1 with hTERT in between S-phase in A549 cell line.

**Additional file 5: Table S1. **shRNA sequences used in this study. **Table S2.** Antibodies for western blot (WB), immunofluorescence (IF), Co-immunoprecipitation (Co-IP) and flow cytometry (FCM).

**Additional file 6:** **Table S3.** PinX1 interaction partners were identified using immunoprecipitation followed by mass spectrometry (IP-MS).

**Additional file 7.** Original data for Western blot in this manuscript.

## Data Availability

The dataset supporting the conclusions of this article is included within Additional file 6.

## Abbreviations

NSCLC: Non-small cell lung cancer
LLC: Lewis lung cancer
IR: Ionizing radiation
TME: Tumor microenvironment
ICB: Immune checkpoint blockade
ICD: Immunogenic cell death
TIME: Tumor immune microenvironment
DSBs: Double-strand breaks
RT: Room temperature
dsDNA: Double-stranded DNA
I-IFN: Type I interferon
ESCC: Esophageal squamous cell carcinomas
PE: Plating efficiency
DDR: DNA damage response
Cell: Counting Kit-8 CCK-8
SDS-PAGE: Sulfate–polyacrylamide gel electrophoresis
PB: Peripheral blood
Co-IP: Co-immunoprecipitation
MS: Mass spectrometry
ATM: Ataxia telangiectasia-mutated
MHC-I: Major histocompatibility complex-I
I-CAMS: Intercellular adhesion molecules
Tex: Exhausted CD8^+^ T cells
PinX1: PIN2/TERF1-interacting telomerase inhibitor
RBM10: RNA-binding motif protein 10
CHK1: Checkpoint kinase 1
ATR: Recombinant antitrypsin related protein
DNA-PKcs: DNA-dependent protein kinase catalytic subunit
cGAS: Cyclic GMP-AMP synthase
STING: Stimulator of interferon genes
Tim-3: T cell immunoglobulin mucin 3

## References

[1] Siegel RL, Miller KD, Jemal A. Cancer statistics, 2020. CA Cancer J Clin. 2020;70:7–30. 31912902 10.3322/caac.21590

[2] Zhou Q, Chen M, Jiang O, Pan Y, Hu D, Lin Q, et al. Sugemalimab versus placebo after concurrent or sequential chemoradiotherapy in patients with locally advanced, unresectable, stage III non-small-cell lung cancer in China (GEMSTONE-301): interim results of a randomised, double-blind, multicentre, phase 3 trial - The Lancet Oncology. Lancet Oncol. 2022;23:209–19. 35038429 10.1016/S1470-2045(21)00630-6

[3] Shaverdian N, Lisberg AE, Bornazyan K, Veruttipong D, Goldman JW, Formenti SC, et al. Previous radiotherapy and the clinical activity and toxicity of pembrolizumab in the treatment of non-small-cell lung cancer: a secondary analysis of the KEYNOTE-001 phase 1 trial. Lancet Oncol. 2017;18:895–903. 28551359 10.1016/S1470-2045(17)30380-7 PMC5538772

[4] Spigel DR, Faivre-Finn C, Gray JE, Vicente D, Planchard D, Paz-Ares L, et al. Five-year survival outcomes from the pacific trial: durvalumab after chemoradiotherapy in stage iii non-small-cell lung cancer. J Clin Oncol. 2022;40:1301–11. 35108059 10.1200/JCO.21.01308 PMC9015199

[5] Theelen WSME, Peulen HMU, Lalezari F, van der Noort V, de Vries JF, Aerts JGJV, et al. Effect of pembrolizumab after stereotactic body radiotherapy vs pembrolizumab alone on tumor response in patients with advanced non-small cell lung cancer: results of the PEMBRO-RT phase 2 randomized clinical trial. JAMA Oncol. 2019;5:1276–82. 31294749 10.1001/jamaoncol.2019.1478 PMC6624814

[6] Galluzzi L, Aryankalayil MJ, Coleman CN, Formenti SC. Emerging evidence for adapting radiotherapy to immunotherapy. Nat Rev Clin Oncol. 2023;20:543–57. 37280366 10.1038/s41571-023-00782-x

[7] Kwon J, Bakhoum SF. The cytosolic DNA-Sensing cGAS-STING pathway in cancer. Cancer Discov. 2020;10:26–39. 31852718 10.1158/2159-8290.CD-19-0761 PMC7151642

[8] Kornepati AVR, Rogers CM, Sung P, Curiel TJ. The complementarity of DDR, nucleic acids and anti-tumour immunity. Nature. 2023;619:475–86. 37468584 10.1038/s41586-023-06069-6

[9] Rossiello F, Jurk D, Passos JF, d’Adda di Fagagna F. Telomere dysfunction in ageing and age-related diseases. Nat Cell Biol 2022; 24: 135–147. 10.1038/s41556-022-00842-x PMC8985209 35165420

[10] Berardinelli F, Coluzzi E, Sgura A, Antoccia A. Targeting telomerase and telomeres to enhance ionizing radiation effects in in vitro and in vivo cancer models. Mutat Res Rev Mutat Res. 2017;773:204–19. 28927529 10.1016/j.mrrev.2017.02.004

[11] Gao J, Pickett HA. Targeting telomeres: advances in telomere maintenance mechanism-specific cancer therapies. Nat Rev Cancer. 2022;22:515–32. 35790854 10.1038/s41568-022-00490-1

[12] Shim G, Ricoul M, Hempel WM, Azzam EI, Sabatier L. Crosstalk between telomere maintenance and radiation effects: a key player in the process of radiation-induced carcinogenesis. Mutat Res Rev Mutat Res 2014; : S1383–5742(14)00002–7. 10.1016/j.mrrev.2014.01.001 PMC4119099 24486376

[13] Saleh AH, Samuel N, Juraschka K, Saleh MH, Taylor MD, Fehlings MG. The biology of ependymomas and emerging novel therapies. Nat Rev Cancer. 2022;22:208–22. 35031778 10.1038/s41568-021-00433-2

[14] Guterres AN, Villanueva J. Targeting telomerase for cancer therapy. Oncogene. 2020;39:5811–24. 32733068 10.1038/s41388-020-01405-w PMC7678952

[15] Ding X, Cheng J, Pang Q, Wei X, Zhang X, Wang P, et al. BIBR1532, a selective telomerase inhibitor, enhances radiosensitivity of non-small cell lung cancer through increasing telomere dysfunction and ATM/CHK1 inhibition. Int J Radiat Oncol Biol Phys. 2019;105:861–74. 31419512 10.1016/j.ijrobp.2019.08.009

[16] Qian D, Zhang B, He L-R, Cai M-Y, Mai S-J, Liao Y-J, et al. The telomere/telomerase binding factor PinX1 is a new target to improve the radiotherapy effect of oesophageal squamous cell carcinomas. J Pathol. 2013;229:765–74. 23341363 10.1002/path.4163

[17] Johnson FB. PinX1 the tail on the chromosome. J Clin Invest. 2011;121:1242–4. 21436580 10.1172/JCI57024 PMC3069792

[18] Cheung DHC, Kung H-F, Huang J-J, Shaw P-C. PinX1 is involved in telomerase recruitment and regulates telomerase function by mediating its localization. FEBS Lett. 2012;586:3166–71. 22749911 10.1016/j.febslet.2012.06.028

[19] Yoo JE, Park YN, Oh B-K. PinX1, a telomere repeat-binding factor 1 (TRF1)-interacting protein, maintains telomere integrity by modulating TRF1 homeostasis, the process in which human telomerase reverse Transcriptase (hTERT) plays dual roles. J Biol Chem. 2014;289:6886–98. 24415760 10.1074/jbc.M113.506006 PMC3945350

[20] You S, Li R, Park D, Xie M, Sica GL, Cao Y, et al. Disruption of STAT3 by niclosamide reverses radioresistance of human lung cancer. Mol Cancer Ther. 2014;13:606–16. 24362463 10.1158/1535-7163.MCT-13-0608 PMC3964811

[21] Yum S, Li M, Chen ZJ. Old dogs, new trick: classic cancer therapies activate cGAS. Cell Res. 2020;30:639–48. 32541866 10.1038/s41422-020-0346-1 PMC7395767

[22] Zhang X, Zhang H, Zhang J, Yang M, Zhu M, Yin Y, et al. The paradoxical role of radiation-induced cGAS-STING signalling network in tumour immunity. Immunology. 2023;168:375–88. 36217274 10.1111/imm.13592

[23] Storozynsky Q, Hitt MM. The impact of radiation-induced DNA damage on cGAS-STING-mediated immune responses to cancer. Int J Mol Sci. 2020;21:8877. 33238631 10.3390/ijms21228877 PMC7700321

[24] Du S-S, Chen G-W, Yang P, Chen Y-X, Hu Y, Zhao Q-Q, et al. Radiation therapy promotes hepatocellular carcinoma immune cloaking via PD-L1 upregulation induced by cGAS-STING activation. Int J Radiat Oncol Biol Phys. 2022;112:1243–55. 34986380 10.1016/j.ijrobp.2021.12.162

[25] Storozynsky Q, Hitt MM. The impact of radiation-induced DNA damage on cGAS-STING-mediated immune responses to cancer. IJMS. 2020;21:8877. 33238631 10.3390/ijms21228877 PMC7700321

[26] Xu L, Zou C, Zhang S, Chu TSM, Zhang Y, Chen W, et al. Reshaping the systemic tumor immune environment (STIE) and tumor immune microenvironment (TIME) to enhance immunotherapy efficacy in solid tumors. J Hematol Oncol. 2022;15:87. 35799264 10.1186/s13045-022-01307-2 PMC9264569

[27] Ozpiskin OM, Zhang L, Li JJ. Immune targets in the tumor microenvironment treated by radiotherapy. Theranostics. 2019;9:1215–31. 30867826 10.7150/thno.32648 PMC6401500

[28] Zhang Z, Liu X, Chen D, Yu J. Radiotherapy combined with immunotherapy: the dawn of cancer treatment. Signal Transduct Target Ther. 2022;7:258. 35906199 10.1038/s41392-022-01102-y PMC9338328

[29] Charpentier M, Spada S, Van Nest SJ, Demaria S. Radiation therapy-induced remodeling of the tumor immune microenvironment. Semin Cancer Biol. 2022;86:737–47. 35405340 10.1016/j.semcancer.2022.04.003

[30] Inoue A. RBM10: Structure, functions, and associated diseases. Gene. 2021;783: 145463. 33515724 10.1016/j.gene.2021.145463 PMC10445532

[31] Chen Y, Deng Z, Jiang S, Hu Q, Liu H, Songyang Z, et al. Human cells lacking coilin and Cajal bodies are proficient in telomerase assembly, trafficking and telomere maintenance. Nucleic Acids Res. 2015;43:385–95. 25477378 10.1093/nar/gku1277 PMC4288172

[32] Venteicher AS, Abreu EB, Meng Z, McCann KE, Terns RM, Veenstra TD, et al. A human telomerase holoenzyme protein required for Cajal body localization and telomere synthesis. Science. 2009;323:644–8. 19179534 10.1126/science.1165357 PMC2728071

[33] Cristofari G, Adolf E, Reichenbach P, Sikora K, Terns RM, Terns MP, et al. Human telomerase RNA accumulation in Cajal bodies facilitates telomerase recruitment to telomeres and telomere elongation. Mol Cell. 2007;27:882–9. 17889662 10.1016/j.molcel.2007.07.020

[34] Ho S-T, Jin R, Cheung DHC, Huang J-J, Shaw P-C. The PinX1/NPM interaction associates with hTERT in early-S phase and facilitates telomerase activation. Cell Biosci. 2019;9:47. 31210926 10.1186/s13578-019-0306-y PMC6567508

[35] Yu C, Chen F, Wang X, Cai Z, Yang M, Zhong Q, et al. Pin2 telomeric repeat factor 1-interacting telomerase inhibitor 1 (PinX1) inhibits nasopharyngeal cancer cell stemness: implication for cancer progression and therapeutic targeting. J Exp Clin Cancer Res. 2020;39:31. 32028978 10.1186/s13046-020-1530-3 PMC7006127

[36] Kang J, Park J-H, Kong JS, Kim MJ, Lee S-S, Park S, et al. PINX1 promotes malignant transformation of thyroid cancer through the activation of the AKT/MAPK/β-catenin signaling pathway. Am J Cancer Res. 2021;11:5485–95. 34873474 PMC8640828

[37] Tian X-P, Jin X-H, Li M, Huang W-J, Xie D, Zhang J-X. The depletion of PinX1 involved in the tumorigenesis of non-small cell lung cancer promotes cell proliferation via p15/cyclin D1 pathway. Mol Cancer. 2017;16:74. 28372542 10.1186/s12943-017-0637-4 PMC5379637

[38] Wang S, Zhang H, Zhu J, Li C, Zhu J, Shi B, et al. PinX1 is a potential prognostic factor for non-small-cell lung cancer and inhibits cell proliferation and migration. Biomed Res Int. 2017;2017:7956437. 28815183 10.1155/2017/7956437 PMC5549499

[39] Ross GM. Induction of cell death by radiotherapy. Endocr Relat Cancer. 1999;6:41–4. 10732785 10.1677/erc.0.0060041

[40] Clark AK, Staniland AA, Marchand F, Kaan TKY, McMahon SB, Malcangio M. P2X7-dependent release of interleukin-1beta and nociception in the spinal cord following lipopolysaccharide. J Neurosci. 2010;30:573–82. 20071520 10.1523/JNEUROSCI.3295-09.2010 PMC2880485

[41] Wan C, Sun Y, Tian Y, Lu L, Dai X, Meng J, et al. Irradiated tumor cell-derived microparticles mediate tumor eradication via cell killing and immune reprogramming. Sci Adv. 2020;6:eaay9789. 32232155 10.1126/sciadv.aay9789 PMC7096163

[42] Mender I, Zhang A, Ren Z, Han C, Deng Y, Siteni S, et al. Telomere stress potentiates STING-dependent anti-tumor immunity. Cancer Cell. 2020;38:400-411.e6. 32619407 10.1016/j.ccell.2020.05.020 PMC7494563

[43] Berardinelli F, Coluzzi E, Sgura A, Antoccia A. Targeting telomerase and telomeres to enhance ionizing radiation effects in in vitro and in vivo cancer models. Mutation Research/Reviews in Mutation Research. 2017;773:204–19. 28927529 10.1016/j.mrrev.2017.02.004

[44] Decout A, Katz JD, Venkatraman S, Ablasser A. The cGAS-STING pathway as a therapeutic target in inflammatory diseases. Nat Rev Immunol. 2021;21:548–69. 33833439 10.1038/s41577-021-00524-z PMC8029610

[45] Byrne A, Savas P, Sant S, Li R, Virassamy B, Luen SJ, et al. Tissue-resident memory T cells in breast cancer control and immunotherapy responses. Nat Rev Clin Oncol. 2020;17:341–8. 32112054 10.1038/s41571-020-0333-y

[46] Jie X, Chen Y, Zhao Y, Yang X, Xu Y, Wang J, et al. Targeting KDM4C enhances CD8+ T cell mediated antitumor immunity by activating chemokine CXCL10 transcription in lung cancer. J Immunother Cancer. 2022;10: e003716. 35121645 10.1136/jitc-2021-003716 PMC8819819

[47] Li X, Gruosso T, Zuo D, Omeroglu A, Meterissian S, Guiot M-C, et al. Infiltration of CD8+ T cells into tumor cell clusters in triple-negative breast cancer. Proc Natl Acad Sci U S A. 2019;116:3678–87. 30733298 10.1073/pnas.1817652116 PMC6397588

[48] Wherry EJ. T cell exhaustion. Nat Immunol. 2011;12:492–9. 21739672 10.1038/ni.2035

[49] Pauken KE, Wherry EJ. Overcoming T cell exhaustion in infection and cancer. Trends Immunol. 2015;36:265–76. 25797516 10.1016/j.it.2015.02.008 PMC4393798

[50] Sakuishi K, Apetoh L, Sullivan JM, Blazar BR, Kuchroo VK, Anderson AC. Targeting Tim-3 and PD-1 pathways to reverse T cell exhaustion and restore anti-tumor immunity. J Exp Med. 2010;207:2187–94. 20819927 10.1084/jem.20100643 PMC2947065

[51] Chow A, Perica K, Klebanoff CA, Wolchok JD. Clinical implications of T cell exhaustion for cancer immunotherapy. Nat Rev Clin Oncol. 2022;19:775–90. 36216928 10.1038/s41571-022-00689-z PMC10984554

[52] Kim J, Bae J-S. Tumor-associated macrophages and neutrophils in tumor microenvironment. Mediators Inflamm. 2016;2016:6058147. 26966341 10.1155/2016/6058147 PMC4757693

[53] Sica A, Larghi P, Mancino A, Rubino L, Porta C, Totaro MG, et al. Macrophage polarization in tumour progression. Semin Cancer Biol. 2008;18:349–55. 18467122 10.1016/j.semcancer.2008.03.004

[54] Jarosz-Biej M, Smolarczyk R, Cichoń T, Kułach N. Tumor microenvironment as a “game changer” in cancer radiotherapy. Int J Mol Sci. 2019;20:3212. 31261963 10.3390/ijms20133212 PMC6650939

[55] Xiao W, Chen X, Li X, Deng K, Liu H, Ma J, et al. RBM10 regulates human TERT gene splicing and inhibits pancreatic cancer progression. Am J Cancer Res. 2021;11:157–70. 33520366 PMC7840715

[56] Jády BE, Richard P, Bertrand E, Kiss T. Cell cycle-dependent recruitment of telomerase RNA and Cajal bodies to human telomeres. Mol Biol Cell. 2006;17:944–54. 16319170 10.1091/mbc.E05-09-0904 PMC1356602

[57] Schmidt JC, Cech TR. Human telomerase: biogenesis, trafficking, recruitment, and activation. Genes Dev. 2015;29:1095–105. 26063571 10.1101/gad.263863.115 PMC4470279

[58] Tomlinson RL, Ziegler TD, Supakorndej T, Terns RM, Terns MP. Cell cycle-regulated trafficking of human telomerase to telomeres. Mol Biol Cell. 2006;17:955–65. 16339074 10.1091/mbc.E05-09-0903 PMC1356603

[59] Aramburu T, Kelich J, Rice C, Skordalakes E. POT1-TPP1 binding stabilizes POT1, promoting efficient telomere maintenance. Comput Struct Biotechnol J. 2022;20:675–84. 35140887 10.1016/j.csbj.2022.01.005 PMC8803944

[60] Sekne Z, Ghanim GE, van Roon A-MM, Nguyen THD. Structural basis of human telomerase recruitment by TPP1-POT1. Science. 2022;375:1173–6. 35201900 10.1126/science.abn6840 PMC7612489

