# Correction to: Silencing PinX1 enhances radiosensitivity and antitumor-immunity of radiotherapy in non-small cell lung cancer

**DOI:** 10.1186/s12967-024-05209-4

**Published:** 2024-05-02

**Authors:** Jieping Qiu, Ying Xia, Yawei Bao, Jingjing Cheng, Lei Liu, Dong Qian

**Affiliations:** 1 Department of Radiation Oncology, The First Affiliated Hospital of USTC, Division of Life Sciences and Medicine, University of Science and Technology of China, Hefei, Anhui China; 2 Core Facility Center, The First Affiliated Hospital of USTC, Division of Life Sciences and Medicine, University of Science and Technology of China, Hefei, Anhui China

**Correction to: Journal of Translational Medicine (2024) 22:228**

10.1186/s12967-024-05023-y

Following publication of the original article [1], we have been notified that affiliations 1 and 3 as well as Funding note were not published correctly.

**It is now:**

1 Present Address: Division of Life Sciences and Medicine, Department of Radiation Oncology, The First Affiliated Hospital of USTC, University of Science and Technology of China, Hefei 230,001, China.

2 Core Facility Center for Medical Sciences, The First Affiliated Hospital of USTC (Anhui Provincial Hospital), Hefei 230,001, Anhui, China.

**Funding**

This study was supported by the National Nature Science Foundation of China (81,773,235 and 81,972,844), and the Fundamental Research Funds for the Central Universities (YD9110000160).

**It should be:**

1 Department of Radiation Oncology, The First Affiliated Hospital of USTC, Division of Life Sciences and Medicine, University of Science and Technology of China, Hefei, Anhui, China.

2 Core Facility Center, The First Affiliated Hospital of USTC, Division of Life Sciences and Medicine, University of Science and Technology of China, Hefei, Anhui, China.

**Funding**

This study was supported by the National Nature Science Foundation of China (81,773,235, 81,972,844), and the Fundamental Research Funds for the Central Universities (YD9100002031 and WK9110000160).

