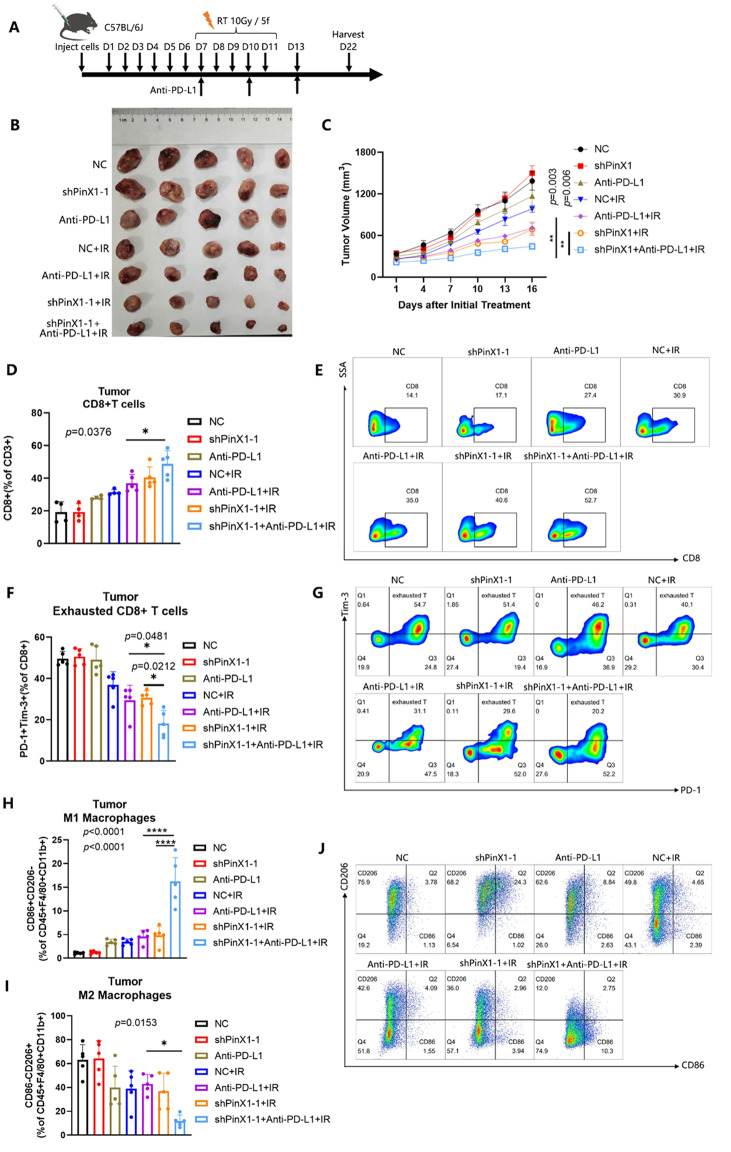# Correction: Silencing PinX1 enhances radiosensitivity and antitumor-immunity of radiotherapy in non-small cell lung cancer

**DOI:** 10.1186/s12967-025-07009-w

**Published:** 2025-09-25

**Authors:** Jieping Qiu, Ying Xia, Yawei Bao, Jingjing Cheng, Lei Liu, Dong Qian

**Affiliations:** 1 Department of Radiation Oncology, The First Affiliated Hospital of USTC, Division of Life Sciences and Medicine, University of Science and Technology of China, Hefei, Anhui China; 2 Core Facility Center, The First Affiliated Hospital of USTC, Division of Life Sciences and Medicine, University of Science and Technology of China, Hefei, Anhui China

**Correction: J Transl Med 22, 228 (2024)** 10.1186/s12967-024-05023-y

Following the publication of the original article, the authors identified errors in Fig. 1G-H, Figs. 3D-E and 4C.

In the originally published version, the survival curves shown in Figs. 1H and 3E were generated based on data recorded starting from day 22, which marked the endpoint of tumor volume monitoring, whereas the tumor volume growth curves began from day 7. This discrepancy in the starting time points led to inconsistencies in data interpretation. A similar issue was present in Fig. 4C.

To address this, the survival data were reanalyzed by setting day 7 post tumor cell inoculation as the starting point, to align with the timeline used for tumor growth monitoring. The survival curves and the x-axis labels of both the survival and tumor volume growth curves in Figs. 1G-H, 3D-E and 4C have been updated accordingly.

These corrections do not affect the study’s results, data interpretation, or conclusions.

The incorrect and correct figures are included in this article.

**The incorrect version of Fig. 1 was**:

**Figure:**
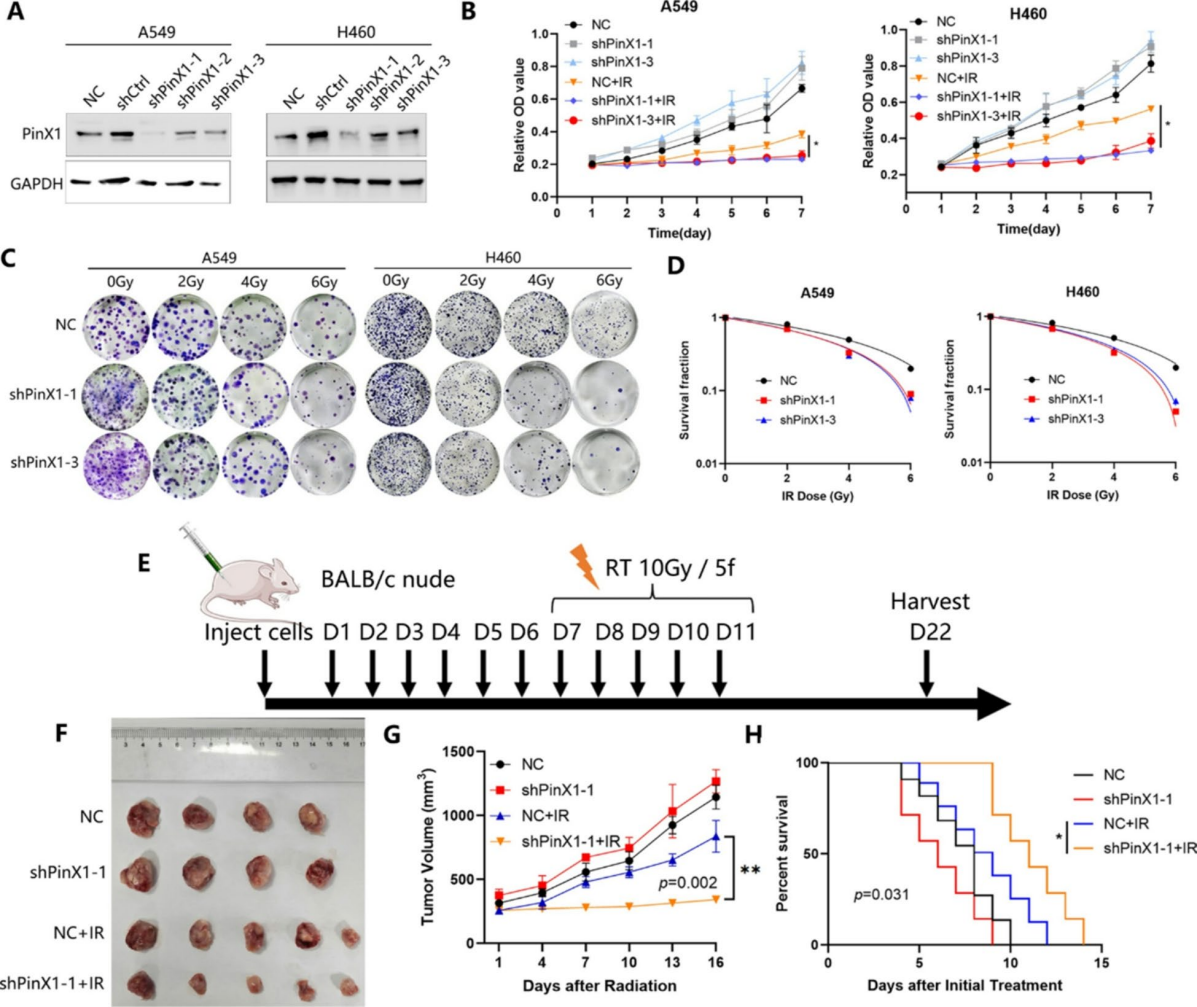

**The correct Fig. 1 is**:

**Figure:**
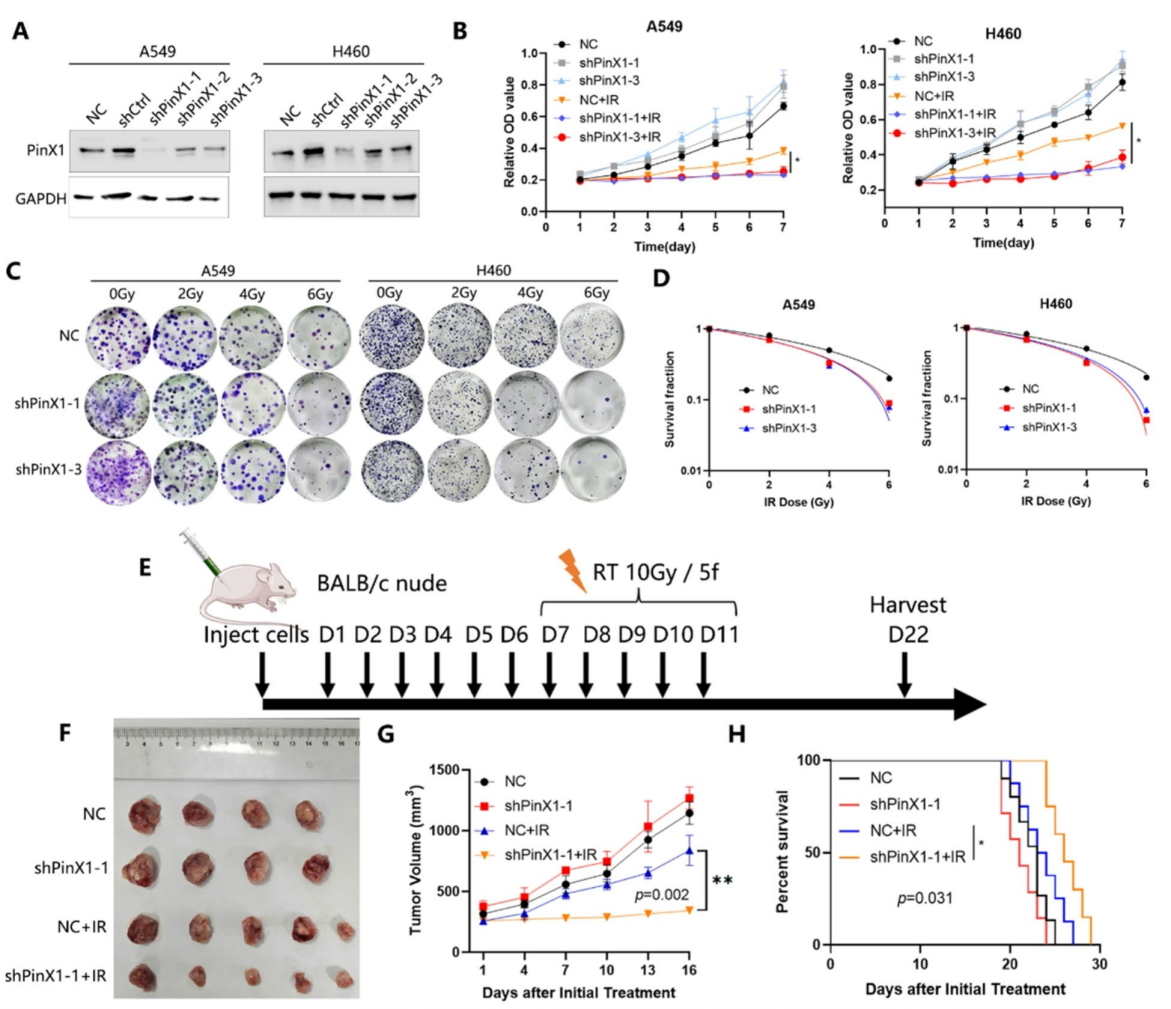

**The incorrect version of Fig. 3 was**:

**Figure:**
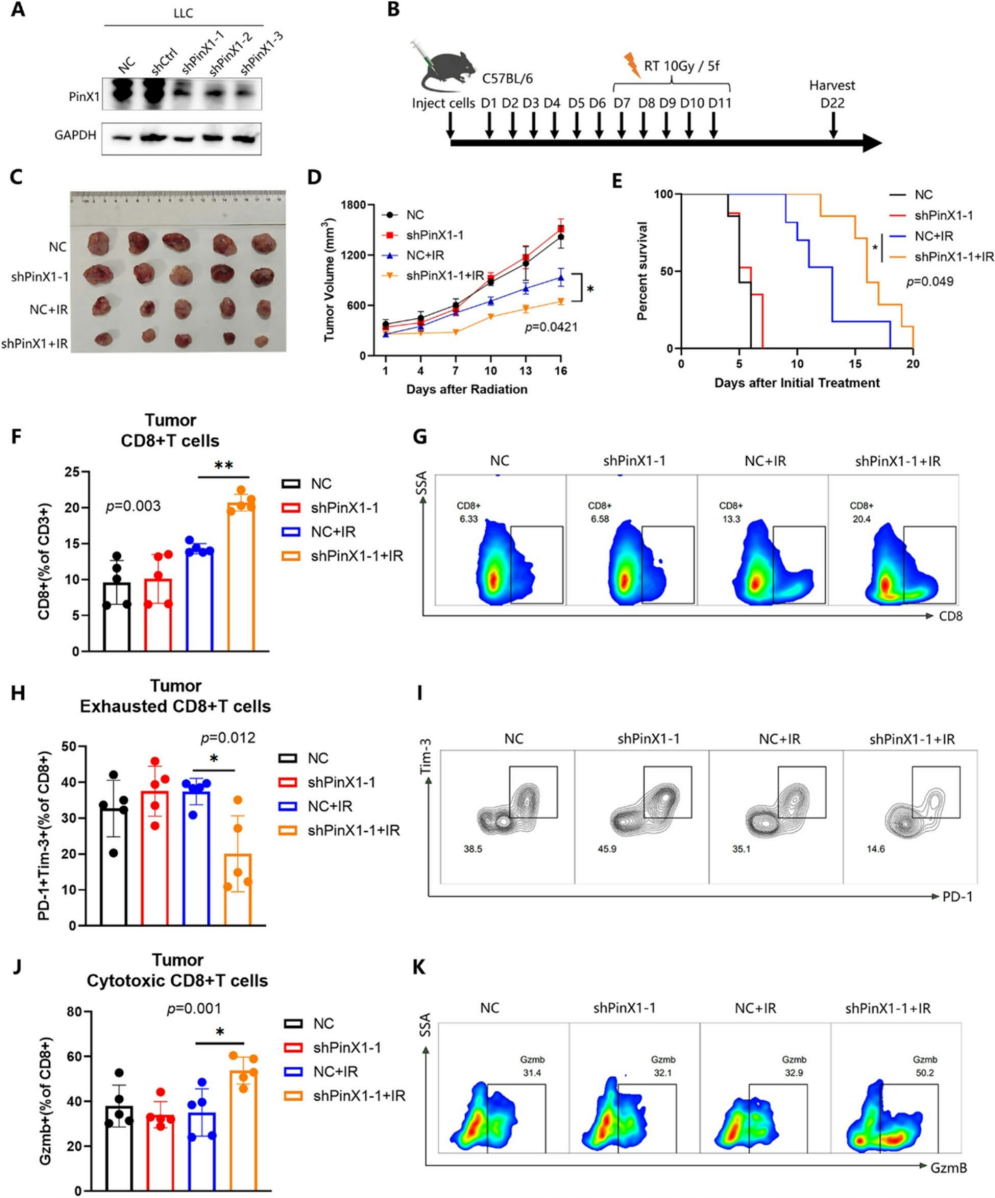

**The correct Fig. 3 is**:

**Figure:**
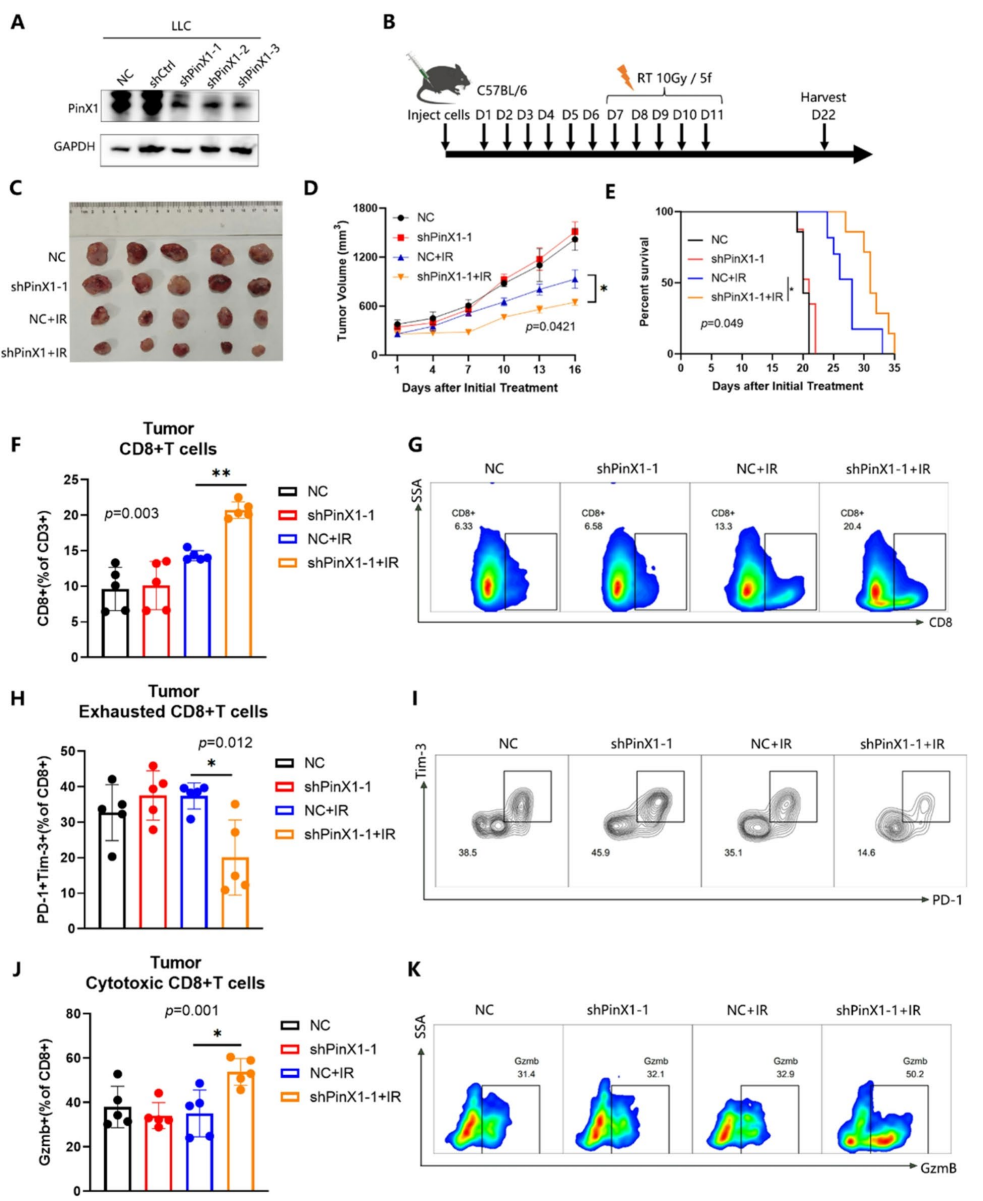

**The incorrect version of Fig. 4 was**:

**Figure:**
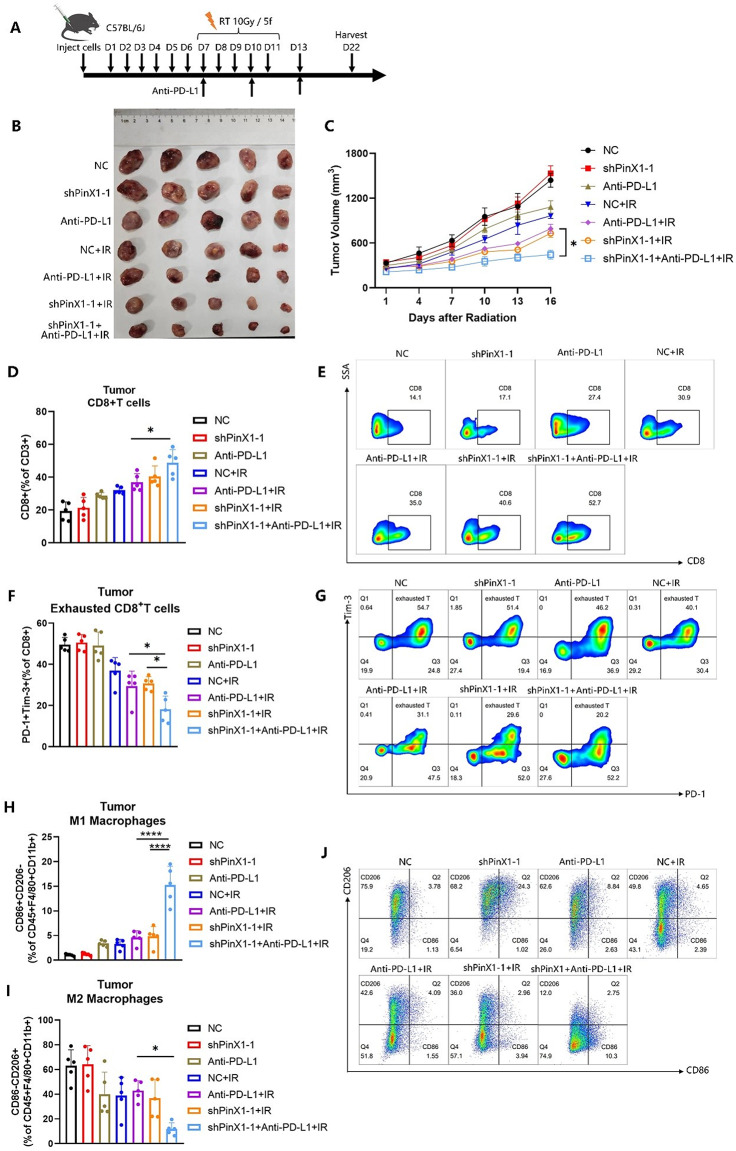

**The correct Fig. 4 is**:

**Figure:**