# Supplementary figures and images for: Silencing PinX1 enhances radiosensitivity and antitumor-immunity of radiotherapy in non-small cell lung cancer
Source: J Transl Med. 2024 Mar 2;22:228. doi: 10.1186/s12967-024-05023-y (PMC10908107; doi:10.1186/s12967-024-05023-y)

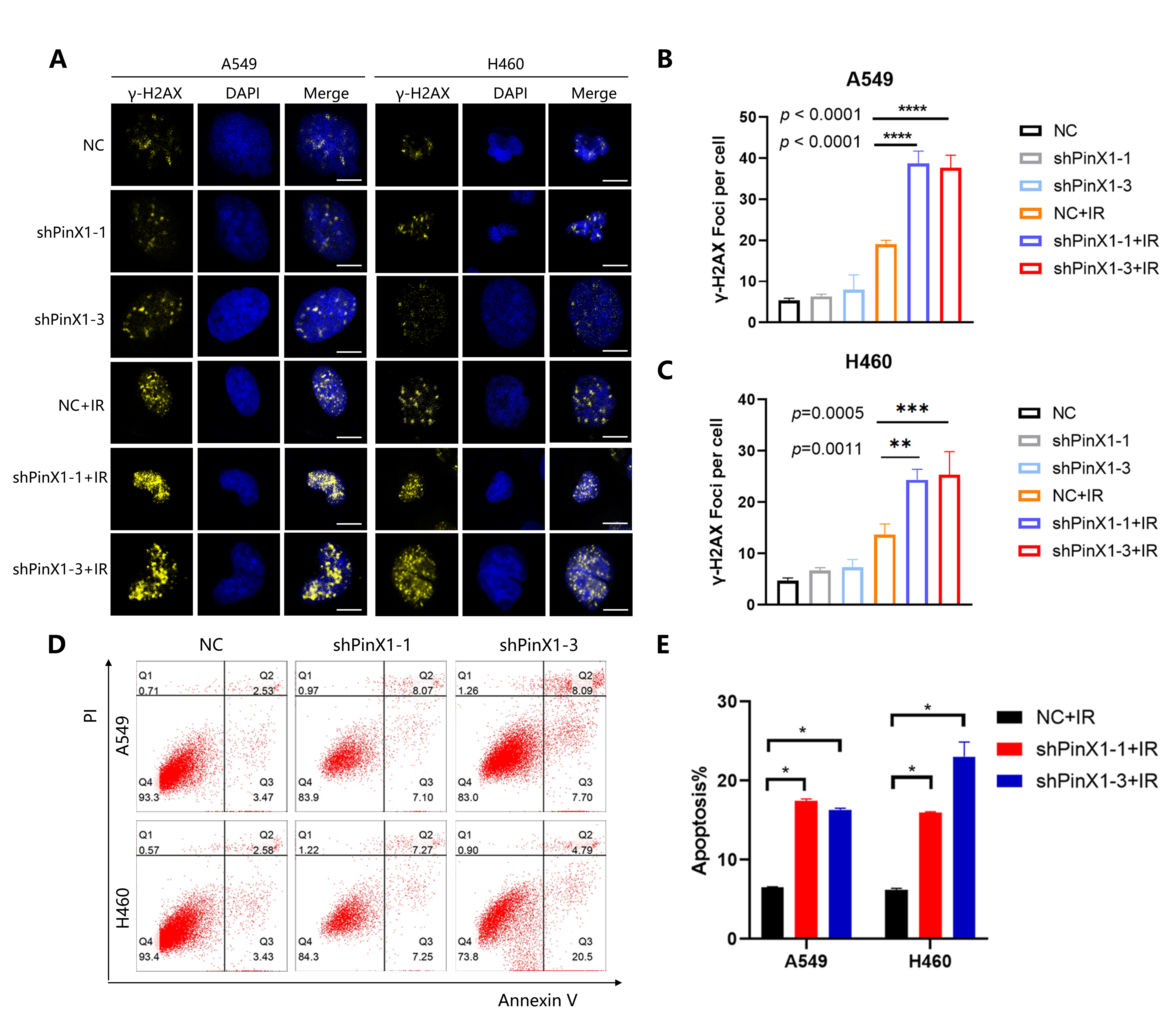

Supplement: Supplementary file 1 — Additional file 1: Fig S1. Silencing of PinX1 enhances IR-induced DNA damage and cell apoptosis. A–C Immunofluorescence was performed to detect γ-H2AX foci formation. Scale bar: 10 μm. n = 3/group. D, E Knockdown of PinX1 enhances IR-induced apoptosis. Forty-eight hours after radiation therapy, cell apoptotic death events were monitored with Annexin V/PI staining and flow cytometry assays. n = 3/group. Data are expressed as the mean ± standard deviation. *: p < 0.05, **: p < 0.01, ***p: < 0.001, ****: p < 0.0001. [file 12967_2024_5023_MOESM1_ESM.png]

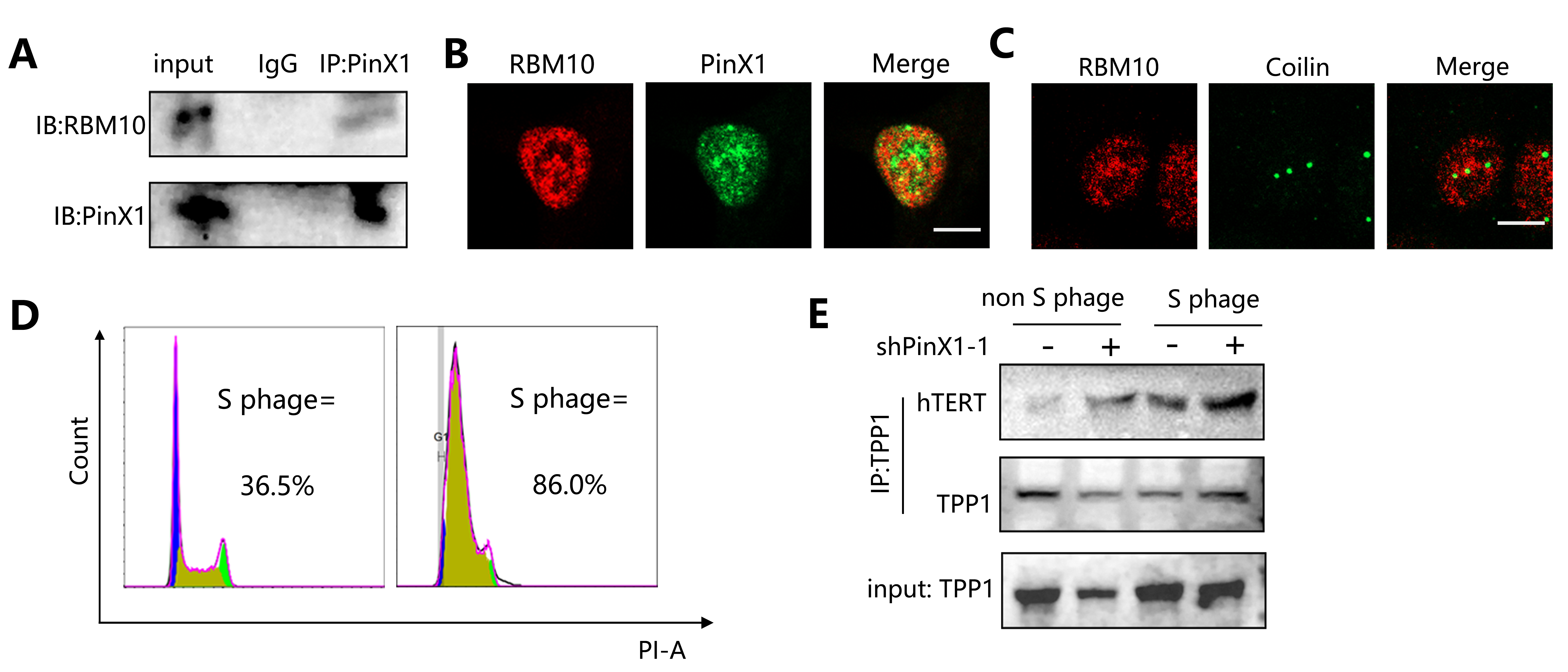

Supplement: Supplementary file 4 — Additional file 4: Fig. S4. PinX1 and TPP1 may synergistically regulate the transport of telomerase to telomeres in different cell cycles. A, B IP and immunofluorescence demonstrated the interaction between PinX1 and RBM10 in A549 cells. Scale bar: 10 μm. C Immunofluorescence proved the co-localization of colin and RBM10 in A549 cells. Scale bar: 10 μm. D After enrichment of S-phase cells by thymidine double blocking, the enrichment efficiency was verified using flow cytometry in A549 cells. E Knockdown of PinX1 enhanced the interaction of TPP1 with hTERT in between S-phase in A549 cell line. [file 12967_2024_5023_MOESM4_ESM.png]
