# Supplementary material for: Silencing PinX1 enhances radiosensitivity and antitumor-immunity of radiotherapy in non-small cell lung cancer
Source: J Transl Med. 2024 Mar 2;22:228. doi: 10.1186/s12967-024-05023-y (PMC10908107; doi:10.1186/s12967-024-05023-y)
Supplement: Supplementary file 5 — Additional file 5: Table S1. shRNA sequences used in this study. Table S2. Antibodies for western blot (WB), immunofluorescence (IF), Co-immunoprecipitation (Co-IP) and flow cytometry (FCM). [file 12967_2024_5023_MOESM5_ESM.docx]

**Additional File 5**

**Table S1: shRNA sequences used in this study**

| shRNA | target sequence |
| --- | --- |
| shPinX1-1 | CACAGATTCCTCGGACAAGAA |
| shPinX1-2 | GCTACACTAGAAGAAACGCTA |
| shPinX1-3 | CAGGTAAAGATGTGGAAAGTT |
| shRBM10-1 | CCCGCAGTCTCAACAAACAAA |
| shRBM10-2 | CAAGACCATCAATGTTGAGTT |
| shRBM10-3 | GACATGGACTACCGTTCATAT |

**Table S2** **Antibodies for western blot (WB),** **immunofluorescence (IF), Co-immunoprecipitation (Co-IP) and flow cytometry (FCM).**

| **Antibodies** | **Concentration** | **Company** | **Clone** | **Application** |
| --- | --- | --- | --- | --- |
| p-ATR (Ser428) | 1:1000 | Cell Signaling Technology | 2853 | WB |
| p-ATM (Ser1981) | 1:1000 | Cell Signaling Technology | 5883 | WB |
| p-CHK1 (Ser345) | 1:1000 | Cell Signaling Technology | 2348 | WB |
| p-DNA-PKcs (S2056) | 1:1000 | abcam | ab18192 | WB |
| GAPDH | 1:1000 | Sangon Biotech | NO.D190090 | WB |
| p-STING (Ser366) | 1:1000 | Cell Signaling Technology | 19781 | WB |
| cGAS | 1:1000 | Proteintech | 26416-1-AP | WB |
| p-TBK1 (Ser172) | 1:1000 | Cell Signaling Technology | 5483 | WB |
| PinX1 | 1:2000 | Proteintech | 12368-1-AP | WB |
| PinX1 | 1:1000 | abcam | ab190252 | WB |
| RBM10 | 1:2000 | abcam | ab72423 | WB |
| Telomerase | 1:1000 | abcam | ab230527 | WB |
| Telomerase | 1:1000 | abcam | ab32020 | WB |
| coilin | 1:500 | abcam | ab87913 | WB |
| tubulin | 1:1000 | abcam | ab56676 | WB |
| anti-Mouse IgG(H+L) | 1:5000 | proteintech | SA00001-1 | WB |
| anti-Rabbit IgG(H+L) | 1:5000 | Proteintech | SA00001-2 | WB |
| γH2AX(Ser139) | 1:2000 | abcam | ab11174 | IF |
| TRF1 | 1:100 | abcam | ab10579 | IF |
| telomerase | 1:50 | abcam | ab230527 | IF |
| coilin | 1:400 | abcam | ab87913 | IF |
| PinX1 | 1:100 | Proteintech | 12368-1-AP | IF |
| RBM10 | 1:500 | abcam | ab220847 | IF |
| DyLight 488 Conjugated AffiniPure Goat Anti-mouse IgG (H+L) | 1:200 | BOSTER | BA1126 | IF |
| DyLight 594 Conjugated AffiniPure Goat Anti-rabbit IgG (H+L) | 1:200 | BOSTER | BA1142 | IF |
| Zombie NIR Fixable Viability Kit | 0.5ul/test | Biolegend | 423106 | FCM |
| CD45 ‒ BV510 | 1ul/test | Biolegend | 103138 | FCM |
| CD3 anti-mouse FITC | 2ul/test | Biolegend | 100204 | FCM |
| Alexa Fluor 700 - CD8a | 2ul/test | Biolegend | 100730 | FCM |
| PerCP/Cyanine5.5 anti-mouse CD4 | 2ul/test | Biolegend | 100540 | FCM |
| CD25 ‒ APC | 2ul/test | Biolegend | 102012 | FCM |
| Foxp3 ‒ PE | 2.5ul/test | Biolegend | 126404 | FCM |
| GzmB ‒ BV421 | 2ul/test | Biolegend | 396414 | FCM |
| CD44 ‒ PE/Cy7 | 2ul/test | Biolegend | 103030 | FCM |
| CD62L ‒ BV421 | 2ul/test | Biolegend | 104436 | FCM |
| PD-1 ‒ PE dazzle594 | 2ul/test | Biolegend | 135205 | FCM |
| Tim3 ‒ BV605 | 2ul/test | Biolegend | 119721 | FCM |
| CD11c ‒ BV421 | 2ul/test | Biolegend | 117330 | FCM |
| MHC II ‒ PE/Cy7 | 2ul/test | Biolegend | 107630 | FCM |
| CD86 ‒ BV605 | 2ul/test | Biolegend | 105125 | FCM |
| CD206 ‒ PE dazzle594 | 2ul/test | Biolegend | 141706 | FCM |
| Telomerase | 1:100 | abcam | ab32020 | Co-IP |
| coilin | 1:10 | abcam | ab87913 | Co-IP |
| PinX1 | 1:100 | abcam | ab190252 | Co-IP |
| TPP1 | 1:50 | Cell Signaling Technology | 14667 | Co-IP |
| RBM10 | 1:10 | abcam | ab72423 | Co-IP |
