# Supplementary material for: Silencing PinX1 enhances radiosensitivity and antitumor-immunity of radiotherapy in non-small cell lung cancer
Source: J Transl Med. 2024 Mar 2;22:228. doi: 10.1186/s12967-024-05023-y (PMC10908107; doi:10.1186/s12967-024-05023-y)

Uncropped Western blot for Fig.1A

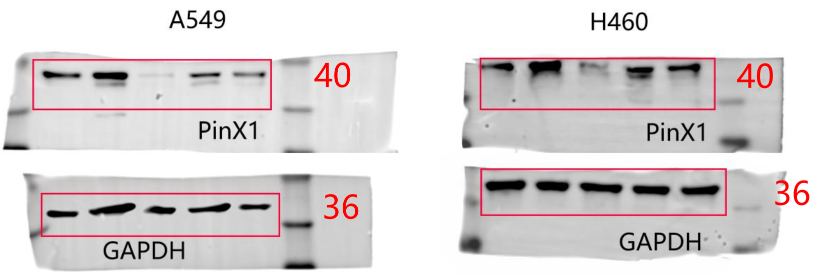

Uncropped Western blot for Fig.2D

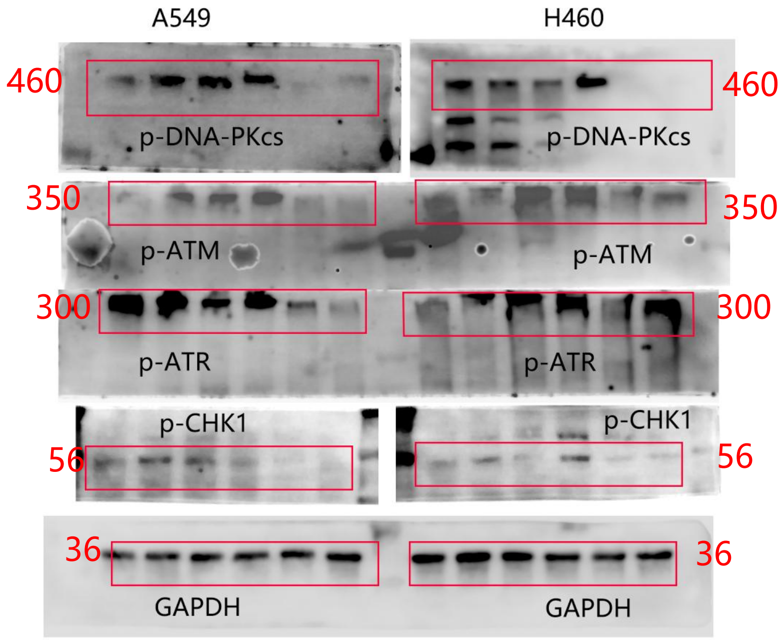

Uncropped Western blot for Fig.2E

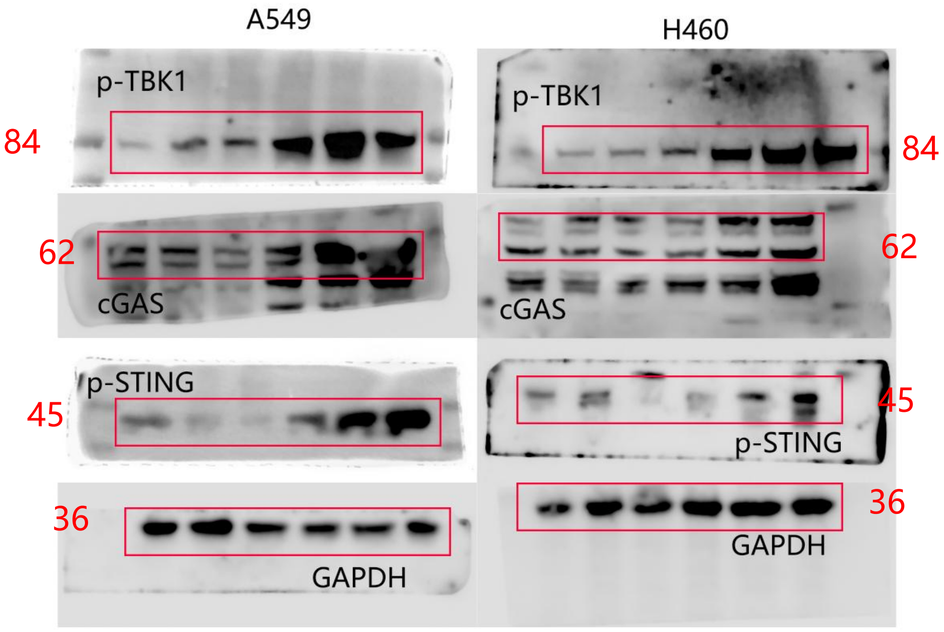

Uncropped Western blot for Fig.3A

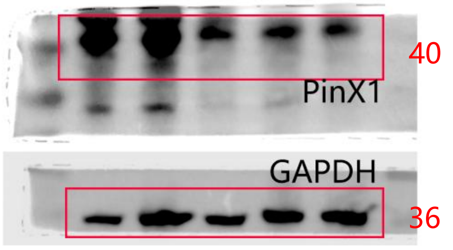

Uncropped Western blot for Fig.5C

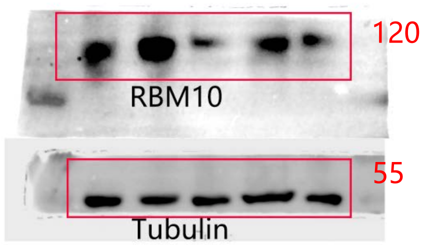

Uncropped Western blot for Fig.5D

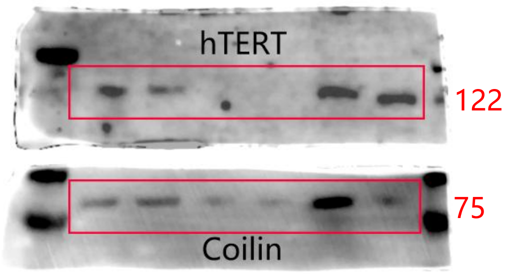

Uncropped Western blot for Fig.5F

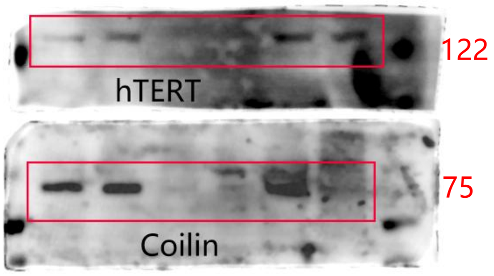

Uncropped Western blot for Fig.5H

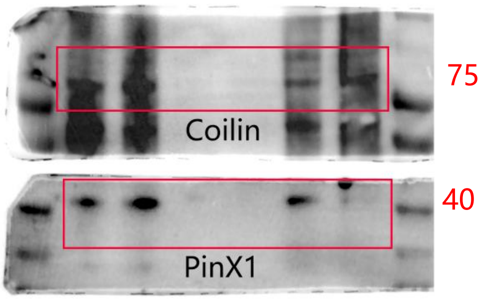

Uncropped Western blot for Fig.S4A

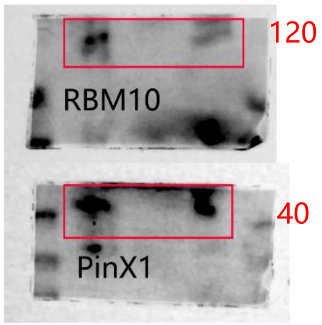

Uncropped Western blot for Fig.S4E

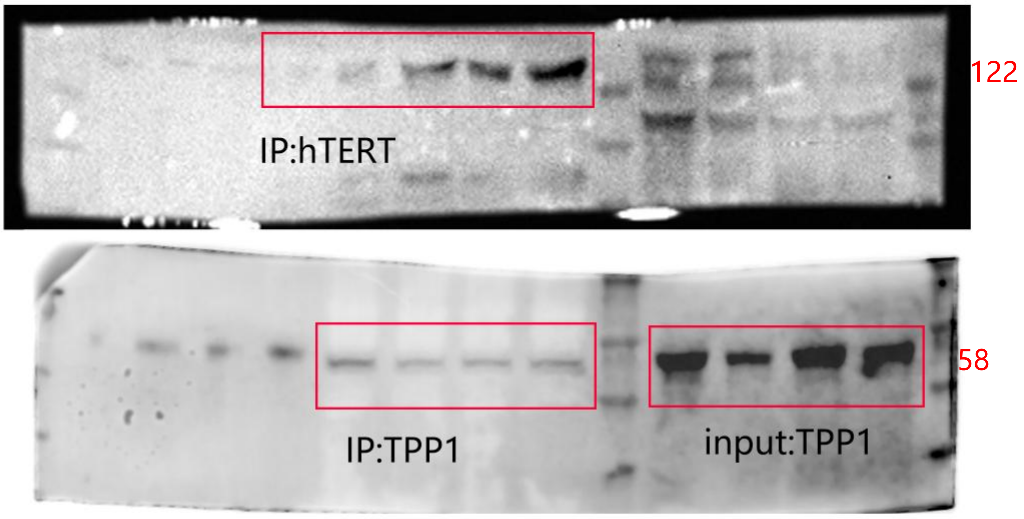

Supplement: Supplementary file 7 — Additional file 7. Original data for Western blot in this manuscript. [file 12967_2024_5023_MOESM7_ESM.pdf]
